# Silicon Enhances Functional Mitochondrial Transfer to Improve Neurovascularization in Diabetic Bone Regeneration

**DOI:** 10.1002/advs.202415459

**Published:** 2025-03-24

**Authors:** Yu‐Xuan Ma, Chen Lei, Tao Ye, Qian‐Qian Wan, Kai‐Yan Wang, Yi‐Na Zhu, Ling Li, Xu‐Fang Liu, Long‐Zhang Niu, Franklin R. Tay, Zhao Mu, Kai Jiao, Li‐Na Niu

**Affiliations:** ^1^ State Key Laboratory of Oral & Maxillofacial Reconstruction and Regeneration National Clinical Research Center for Oral Diseases Shaanxi Key Laboratory of Stomatology Department of Prosthodontics School of Stomatology The Fourth Military Medical University Xi'an 710032 China; ^2^ The Dental College of Georgia Augusta University Augusta GA 30912 USA; ^3^ State Key Laboratory of Oral & Maxillofacial Reconstruction and Regeneration School of Stomatology The Fourth Military Medical University Xi'an 710032 China; ^4^ Department of Stomatology Tangdu hospital State Key Laboratory of Oral & Maxillofacial Reconstruction and Regeneration & National Clinical Research Center for Oral Diseases & Shaanxi Key Laboratory of Stomatology School of Stomatology & Shaanxi Key Laboratory of Stomatology School of Stomatology The Fourth Military Medical University Xi'an Shaanxi 710032 China

**Keywords:** bioactive silicon, diabetic bone defects, macrophages, mitochondrial transfer, neural, vascular

## Abstract

Diabetes mellitus is a metabolic disorder associated with an increased risk of fractures and delayed fracture healing, leading to a higher prevalence of bone defects. Recent advancements in strategies aim at regulating immune responses and enhancing neurovascularization have not met expectations. This study demonstrates that a silicon‐based strategy significantly enhances vascularization and innervation, thereby optimizing the repair of diabetic bone defects. Silicon improves mitochondrial function and modulates mitochondrial fission dynamics in macrophages via the Drp1‐Mff signaling pathway. Subsequently, functional mitochondria are transferred from macrophages to endothelial and neuronal cells through microvesicles, providing a protective mechanism for blood vessels and peripheral nerves during early wound healing. On this basis, an optimized strategy combining a silicified collagen scaffold with a Drp1‐Fis1 interaction inhibitor is used to further regulate mitochondrial fission in macrophages and enhance the trafficking of functional mitochondria into stressed receptor cells. In diabetic mice with critical‐sized calvarial defects, the silicon‐based treatment significantly promotes vessel formation, nerve growth, and mineralized tissue development. These findings provide therapeutic insights into the role of silicon in promoting diabetic bone regeneration and highlight the importance of intercellular communication in diabetic conditions.

## Graphical abstract

These findings reveal that silicon exerts strong protective effects in diabetic bone defect models by selectively promoting Drp1‐Mff activity. Silicon facilitates precise regulation of mitochondrial dynamics to enable functional mitochondrial transfer. The findings demonstrate the effectiveness of silicon in alleviating diabetic bone defects and its potential as an innovative therapeutic approach.

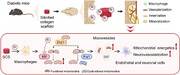

## 1 Introduction

Diabetes mellitus (DM) is a metabolic disorder characterized by hyperglycemia, projected to affect 642 million individuals globally by 2040.^[^ ^1^ ^]^ Patients with DM face substantial challenges in the management of bone quality. Epidemiological evidence confirms that patients with DM have an increased fracture risk and delayed fracture healing, leading to a higher occurrence of bone defects. These complications result in prolonged inactivity and hospitalization, ultimately increasing morbidity and mortality rates.^[^ ^2^, ^3^ ^]^ Unfortunately, most existing bone repair biomaterials are designed for healthy individuals, resulting in limited clinical benefits for patients with DM.^[^ ^4^ ^]^ Moreover, the effects of previous hyperglycemia persist even after optimal glycemic control, a condition known as metabolic memory.^[^ ^5^ ^]^ Because of the long‐lasting detrimental effects on diabetic bone, there is an urgent need to develop new biomaterials to improve diabetic bone regeneration.

Diabetes mellitus has multiple negative effects on the bone tissue. These effects involve the local immune response and neurovascularization. Macrophages play a crucial role in the healing of osseous wounds and in immunomodulatory reactions in response to biomaterials.^[^ ^6^, ^7^ ^]^ However, hyperglycemia causes a delayed and prolonged inflammatory response in diabetic individuals.^[^ ^8^, ^9^ ^]^ Impaired vascularization and innervation are significant features in patients suffering from poor osteoinduction efficacy of implanted biomaterials.^[^ ^10^, ^11^ ^]^ Furthermore, the integrity of diabetic bone is also altered due to the rarefaction of microvasculature^[^ ^12^, ^13^ ^]^ and innervation,^[^ ^14^, ^15^ ^]^ reflecting anatomical changes in diabetic peripheral tissues. Significant strides have been made in the development of biomaterials designed to modulate the immune response and enhance and vascularization to address diabetic bone defects.^[^ ^16^, ^17^ ^]^ However, there remain certain limitations in the current research, particularly with respect to realize innervation using present therapeutic strategies. Additionally, the immune response, vascularization, and innervation are interdependent and collectively influence the regeneration of bone tissue.^[^ ^18^, ^19^ ^]^ A comprehensive understanding of the mechanisms by which these biological processes interact in the context of diabetes is still lacking. Therefore, holistic materials with optimal biological effects are required in diabetic individuals.^[^ ^20^ ^]^ These materials provide the regulatory cues necessary for controlling the fate of macrophages, endothelial cells, and peripheral nerves.

Silicon, as an essential trace element, has garnered attention for its critical role in bone health and repair. It is a functional component of Bio‐Oss (bovine‐derived bone repair material) and Bio‐glass (synthetic bone repair material), both of which are commonly used in the reconstruction of craniofacial and alveolar bone defects. Prior research indicates that silicon‐doped scaffolds activate macrophages^[^ ^21^, ^22^ ^]^ and enhance vascularization and nerve innervation,^[^ ^23^, ^24^ ^]^ which ultimately positively influence osteogenesis. Furthermore, the prolonged diabetic condition results in metabolic abnormalities in nearly all cell types, including macrophages, endothelial cells, and peripheral nerves.^[^ ^25^, ^26^ ^]^ Biomarkers of mitochondrial dysfunction have been proposed to stratify patients at risk of chronic complications associated with diabetes. Silicon is found to activate the antioxidant defense system and regulate the accumulation of reactive oxygen species (ROS), indicating its potential to promote mitochondrial function.^[^ ^27^, ^28^ ^]^ However, most of these studies have been conducted under normal energy metabolism conditions, and whether silicon can improve the regeneration process of bone defects in diabetic conditions under abnormal cellular energy metabolism remains unclear.

In this work, we provide insights into the role of silicon in promoting diabetic bone regeneration and highlight the importance of understanding intercellular communication during diabetic conditions. Our findings indicate that silicon enhances mitochondrial function in macrophages via the Drp1‐Mff pathway, facilitating the transfer of functional mitochondria to endothelial and neuronal cells and restoring their energy metabolism and cell differentiation. Combining silicon with a Drp1‐Fis1 inhibitor reduces mitochondrial damage and further improves the transfer of functional mitochondria. This approach enhances vessel formation, neuronal growth, and mineralized tissue development, offering a promising strategy to improve bone regeneration under diabetic conditions.

## 2 Results

### 2.1 The Effect of Silicon on Diabetic Bone Regeneration

**Figure 1:**
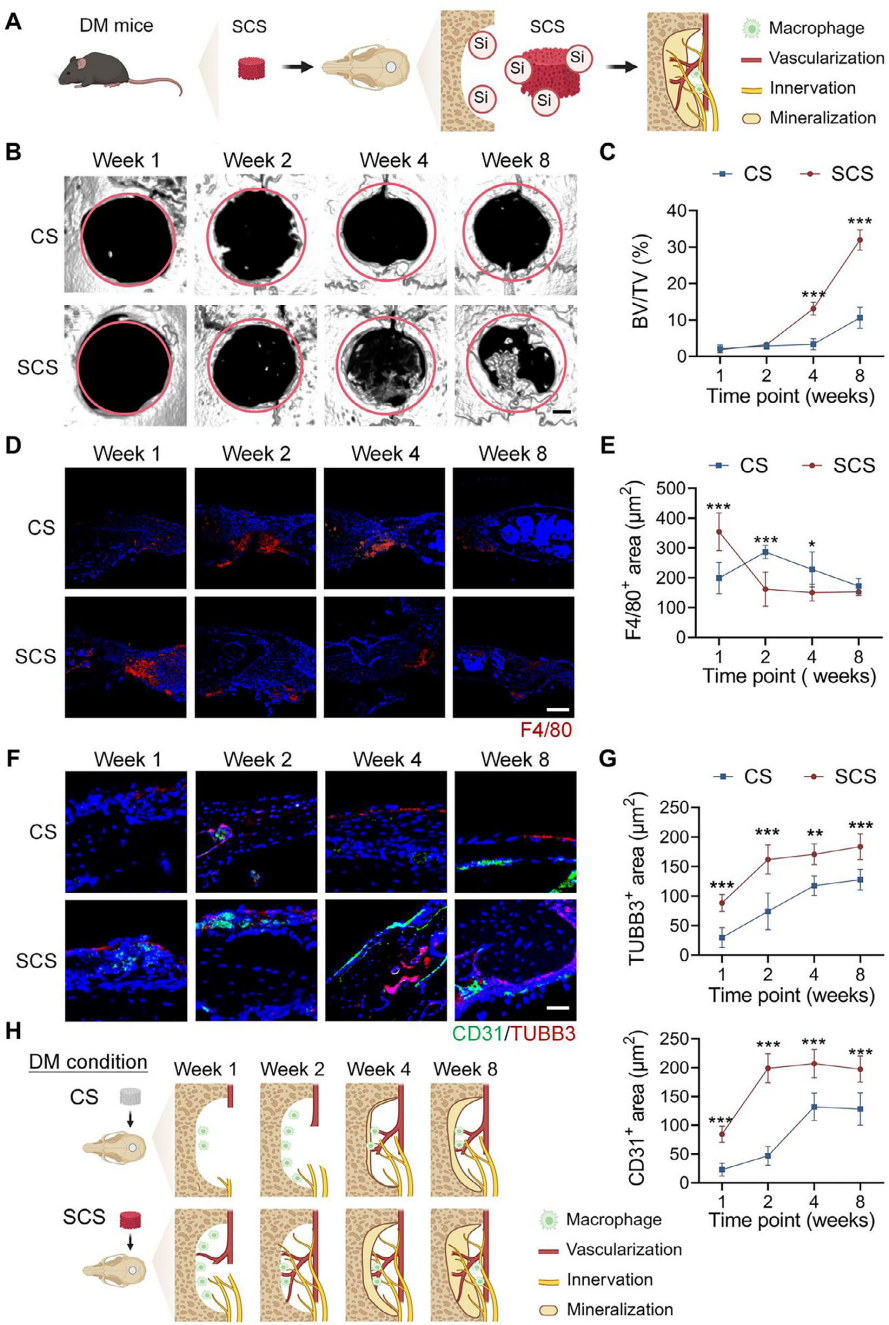
Silicon promotes inflammatory response, vascularization, innervation, and mineralization during diabetic bone repair. A) Illustration of silicon release after SCS implantation in diabetic mice with critical‐sized calvarial defects. The ossification, immune response, and neurovascularization were analyzed using micro‐computed tomography and histological examination. B,C) Representative micro‐CT images and quantitative analysis of newly‐formed bone (*n* = 4). The red‐dotted circle indicates approximately the original defect zone. Scale bar: 500 mm. D,E) Representative stained images and quantification of F4/80^+^ macrophages within the calvarial defect site (*n* = 4). Scale bar: 200 µm. F,G) Representative stained images and quantification of CD31^+^ vessels and TUBB3^+^ nerves within the calvarial defect site (*n *= 4). Scale bar: 100 µm. H) Schematic showing the diabetic bone regeneration after CS or SCS implantation. SCS triggers an earlier immune response, promotes neurovascular regeneration, and leads to improved outcomes during the repair of diabetic bone defects. CS: collagen scaffold, SCS: silicified collagen scaffolds, BV/TV: bone volume/total volume. Statistical analysis was performed using Student's *t*‐test. with significance defined as *P < 0.05, **P < 0.01, ***P < 0.001. All illustrations were created using Gallery software and are available for public utilization.

To investigate the potential role of silicon in the healing of diabetic bone defects, silicified collagen scaffolds (SCS) were used to implant in diabetic mice (**Figure** **1**A). SCS was prepared by condensing silicic acid precursors into a pure collagen scaffold (CS) (Figure S1A, Supporting Information).^[^ ^29^ ^]^ The SCS continuously released a biologically stable source of silicon in the form of fluidic silicic acid (Figure S1B, Supporting Information).^[^ ^30^ ^]^ Both CS and SCS displayed similar periodic microstructures characteristic of collagen fibrils, as well as analogous surface roughness profiles (Figure S1C,D, Supporting Information). Full‐thickness and critical‐sized cranial defects were created in diabetic mice to compare the effects of SCS and CS on bone remodeling over an eight‐week period (Figure S2A, Supporting Information). No significant differences in blood glucose or body weight were observed between the CS and SCS groups (Figure SB,C, Supporting Information). This observation indicated that SCS did not significantly affect the systemic metabolic status of the diabetic mice.

Micro‐computed tomography (micro‐CT) and hematoxylin and eosin staining (H&E) were performed to examine whether SCS promotes in situ ossification. Quantitative analysis of reconstructed micro‐CT images showed no significant difference in ossification between the SCS and CS groups in the first and second weeks (Figure 1B,C). However, the SCS group showed significant improvement in bone healing commencing the fourth week. In addition, H&E staining showed a larger area of newly‐formed osteoid tissue in the SCS group at the eighth week (Figure S3, Supporting Information). Therefore, SCS application enabled more favorable restoration of bone mass in diabetic mice during the latter stages of bone healing.

The immune, vascular, and nervous systems are crucial in the bone repair process,^[^ ^6^, ^7^, ^10^, ^11^ ^]^ raising the question of whether SCS enhances bone healing by influencing these systems. Therefore, immunofluorescence staining and quantification were conducted using cell type‐specific antibodies at different time‐points. We found that the expression of F4/80^+^ macrophages in the SCS group underwent rapid expansion, peaking in the first week, followed by a decrease that later equilibrated, In contrast, the expression level in the CS group did not peak until the second week. Throughout the eight‐week experiment, the densities of CD31^+^ vascular endothelium and TUBB3^+^ nerve fibers in the SCS group were significantly higher than those in the CS group (Figure 1D,E). The SCS group demonstrated a peak in vascular and neuronal density in the second week, whereas the CS group did not reach its peak until the fourth week (Figure 1F,G). These results indicate that silicon triggers an earlier immune response, promotes neurovascular regeneration, and leads to improved outcomes during the repair of diabetic bone defects (Figure 1H). However, the mechanisms by which silicon influences immunity and neurovascularization remain unclear. Research has demonstrated that diabetes can induce metabolic abnormalities in nearly all cell types.^[^ ^25^, ^26^ ^]^ Therefore, it is essential to investigate whether silicon regulates the function of macrophages, endothelial cells, and nerve cells by enhancing their metabolic capacity under diabetic conditions. To this end, we conducted further studies to examine the effects of silicon on the metabolic capacities of macrophages, endothelial cells, and nerve cells in a simulated diabetic environment in vitro.

### 2.2 Effect of Silicon on Cellular Energy Metabolism under Hyperglycemic Stress

**Figure 2:**
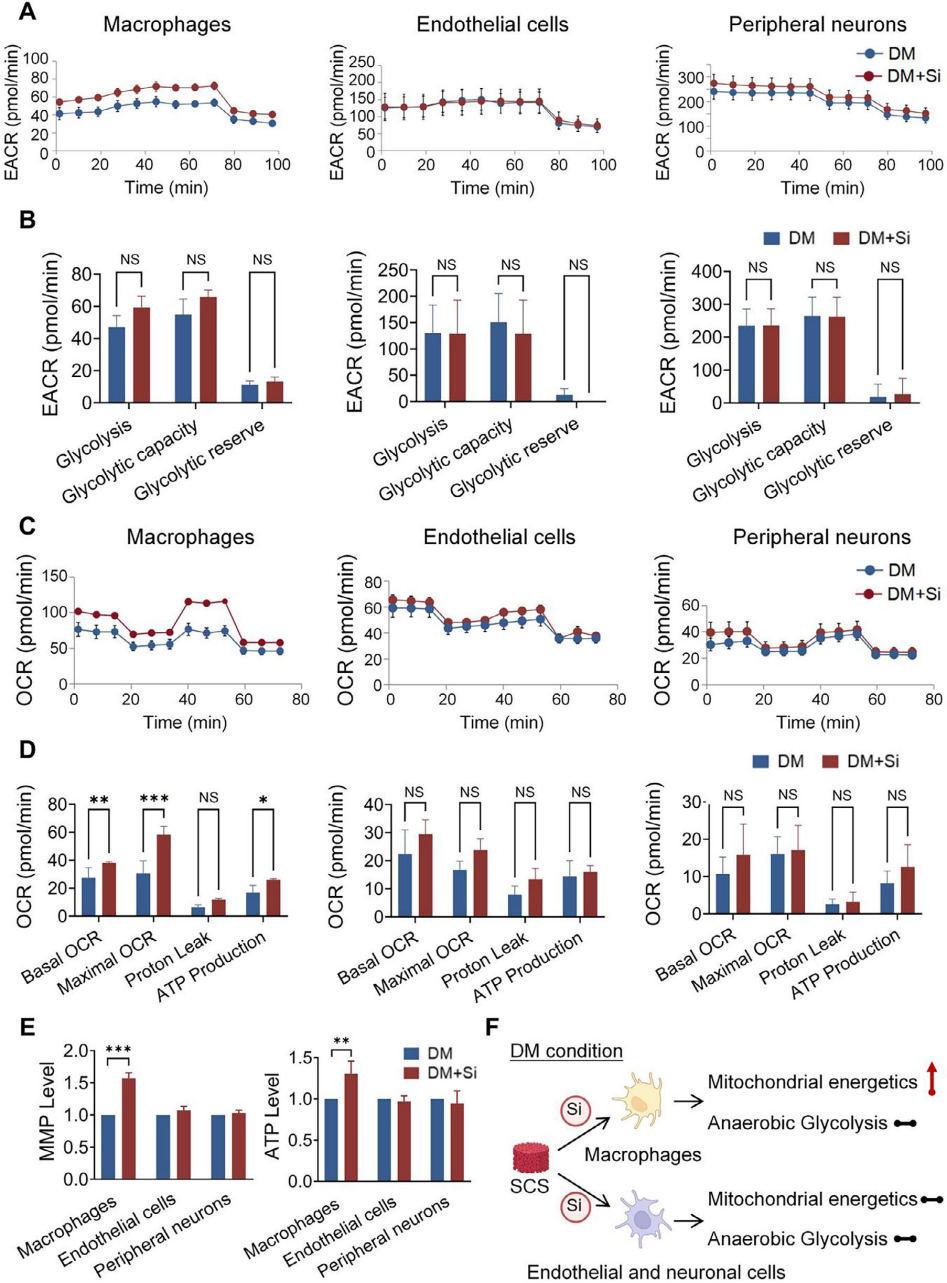
Silicon improves mitochondrial respiration in macrophages, but not in endothelial cells or peripheral neurons under simulated diabetic conditions. A,B) Measurement of EACR, glycolysis, glycolytic capacity, and glycolytic reserve were measured in macrophages, endothelial cells, and neuronal cells, respectively (*n* = 4). C,D) Measurement of OCR, basal respiration, maximal respiration, spare respiratory capacity, and ATP production were measured in macrophages, endothelial cells, and peripheral neurons, respectively (*n *= 4). E) ATP level and MMP level of various cell types under simulated diabetic conditions, in the absence or presence of silicon (*n *= 6). F) Schematic showing the energy metabolism of various cell types under diabetic conditions with silicon supplement. DM: diabetic condition, DM+Si: diabetic condition with silicon supplement, EACR: extracellular acidification rate., OCR: oxygen consumption rate, ATP: adenosine triphosphate, MMP: mitochondrial membrane potential. Statistical analysis was performed using Student's *t‐*test. with significance defined as *P < 0.05, **P < 0.01, ***P < 0.001, NS: not significant. All illustrations were created using Gallery software and are available for public utilization.

To investigate the effect of silicon on energy metabolism in diabetic conditions, a hyperglycemic stress condition was first established to replicate the metabolic stress characteristics of diabetes mellitus in vitro. This condition was examined by measuring cellular proliferation and levels of ROS (Figure S4, Supporting Information). Then, we measured the oxygen consumption rate (OCR) and extracellular acidification rate in macrophages, endothelial cells, and peripheral neurons under this condition. The results suggest that the glycolytic capacity of the three cell types remained unaffected by silicon supplementation, derived from SCS extracts, under hyperglycemic stress conditions (**Figure** **2**A,B). Furthermore, silicon supplementation selectively enhanced mitochondrial oxidative phosphorylation capacity in macrophages under hyperglycemic stress conditions (Figure 2C,D). Conversely, no significant effect was observed in endothelial cells or peripheral neurons. Similarly, mitochondrial function in macrophages subjected to hyperglycemic stress was significantly improved in the presence of silicon (Figure 2E). This was demonstrated by the increase in mitochondrial membrane potential (MMP) levels and adenosine triphosphate (ATP) production. No significant improvement in mitochondrial function was observed in endothelial cells or peripheral neurons. These results suggest that under simulated diabetic conditions, energy metabolism may be modulated by silicon through the enhancement of mitochondrial function in macrophages, but not in endothelial cells and neuronal cells (Figure 2F). The mechanisms by which silicon affects endothelial and neuronal cells remain unclear.

### 2.3 Silicon Influences Neurovascularization through Macrophages‐Mediated Intercellular Communication under Diabetic Condition

**Figure 3:**
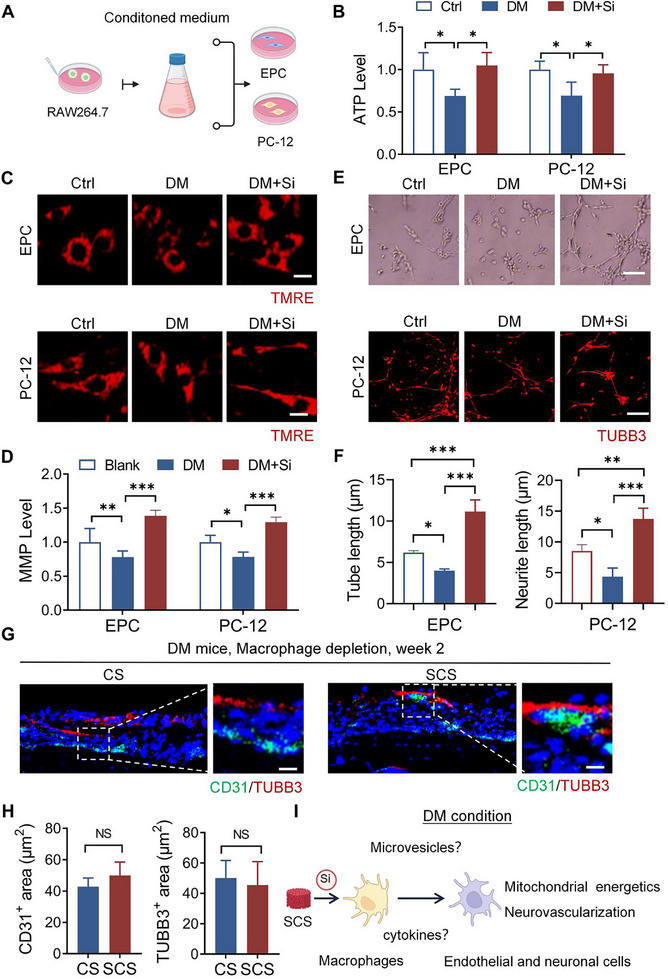
Silicon interacts with macrophages to enhance angiogenesis and nerve regeneration. A) Schematic of the experimental set‐up. B) Quantification of intracellular ATP levels in endothelial progenitor cells (EPC) and neurons (PC‐12) under the indicated treatments (*n* = 6). C,D) Representative images and quantification of mitochondrial membrane potential in EPC and PC‐12 cells using TMRE intensity (*n* = 6). Scale bar: 10 µm. E,F) Representative images and quantification of tubular structures formed by EPC and neurite length in PC‐12 cells under the indicated treatments (*n *= 6). Scale bar: 100 µm. G,H) Representative images and quantification of CD31^+^ vessels and TUBB3^+^ nerve fibers within the calvarial defect site in macrophage‐depleted diabetic mice (*n *= 4). Scale bar: 20 µm. I) Schematic illustrating the influence of macrophages on endothelial and neuronal cells under diabetic conditions. CM: conditioned medium, Blank: blank control condition, DM: diabetic condition, DM+Si: diabetic condition with silicon supplement. Statistical analysis was performed using Student's *t*‐test. with significance defined as ***P < 0.001, NS: not significant. All illustrations were created using Gallery software and are available for public utilization.

There is ample evidence indicating that macrophages guide the regeneration of blood vessels and peripheral nerves through intercellular communication during bone repair.^[^ ^18^, ^19^ ^]^ Consequently, the role of silicon‐activated macrophages in intercellular communication warrants further investigation. To investigate the role of silicon in intercellular communication under diabetic conditions, a conditioned culture medium from macrophages treated with or without silicon was used to assess energy metabolism and cell differentiation in endothelial and neuronal cells (**Figure** **3**A). The levels of adenosine triphosphate (ATP) (Figure 3B) and MMP (Figure 3C,D) were significantly elevated in both the blank control (Blank) group and the diabetic with silicon (DM+Si) group compared to the diabetic (DM) group. Furthermore, tubule formation and axon elongation assays demonstrated significantly enhanced differentiation of endothelial and neuronal cells in the DM+Si group compared to both the DM and Blank groups (Figure 3E,F). The Blank group served as a baseline for comparison. The DM+Si group exhibited metabolic activity and cell differentiation levels comparable to those of the Blank group. These findings suggest that silicon indirectly regulates vascular and neuronal cells through macrophages to influence on diabetic bone regeneration. These findings suggest that silicon may indirectly regulate vascular and neuronal cells through macrophages, thereby exerting a broader influence on diabetic bone regeneration. We further verified this finding in vivo by implanting SCS and CS into the skull defects of macrophage‐depleted diabetic mice (Figure S5, Supporting Information). No significant differences in local neurovascularization were observed between the two groups in the second week. This finding also suggests that silicon promotes vessel and nerve regeneration via macrophages at the early stage (Figure 3G,H).

Collectively, these results suggest that silicon promotes energy metabolism and cell differentiation in diabetes through intercellular communication (Figure 3I). Macrophages can influence adjacent cells through various forms of intercellular communication, including cytokine secretion and the release of microvesicles containing bioactive substances.^[^ ^18^, ^19^ ^]^ However, the specific mechanisms by which silicon participates in macrophage‐mediated intercellular communication require further investigation.

### 2.4 Macrophages Transfer Functional Mitochondria to Endothelial and Neuronal Cells via Microvesicles under Diabetic Condition

**Figure 4:**
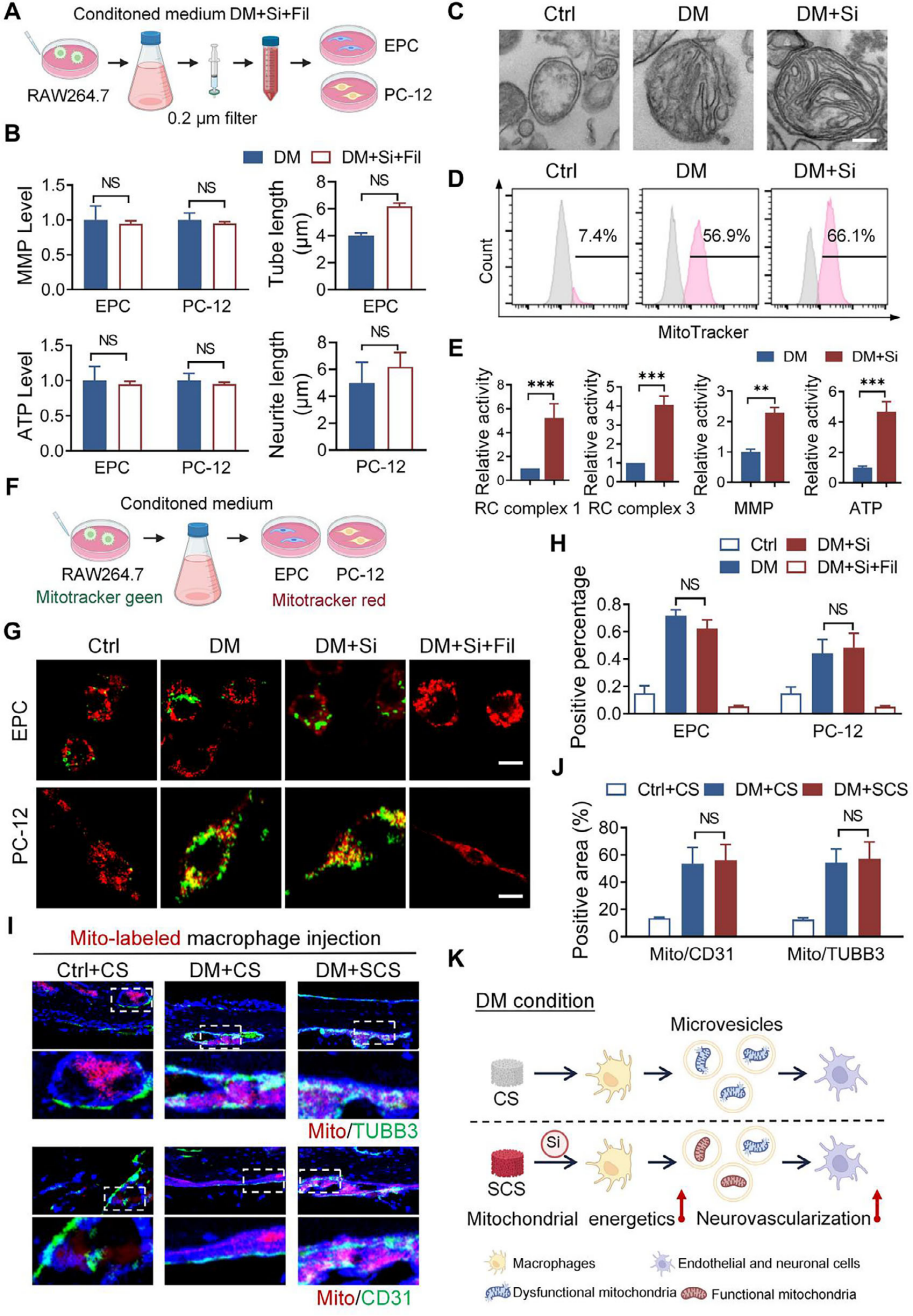
Macrophages transfer mitochondria to endothelial and neuronal cells under simulated diabetic (DM) conditions. A) Schematic showing the experimental set‐up to investigate intercellular communication. B) Quantification of intracellular ATP protection and mitochondrial membrane potential in endothelial progenitor cells (EPC) or PC‐12 under indicated treatments (*n* = 6). C) Representative TEM pictures of extracellular vesicles and extracellular mitochondria derived from the macrophage‐conditioned medium after DM or DM+Si treatment. Scale bar: 100 nm. D) Macrophages were labeled with MitoTracker. FACS showed that extracellular mitochondria were present in the macrophage‐conditioned medium. E) Mitochondrial respiratory chain complex activity, mitochondrial membrane potential, and ATP content in extracellular mitochondria derived from macrophage under DM or DM+Si condition (*n* = 4). F) Schematic showing the experimental set‐up to investigate intercellular mitochondria transfer. G,H) Representative images and quantification of exogenous (green, from macrophages) and endogenous (red) mitochondrial signals observed in EPC and PC‐12 cells (*n *= 6). Scale bar: 10 µm. I,J) Representative images and quantification of Mito‐labeled macrophage (red) co‐localizing with CD31^+^ blood vessels or TUBB3^+^ nerve fibers (green) within the calvarial defect site (*n* = 4). Scale bar: 20 µm. K) Schematic showing that macrophages transfer mitochondria in different functional states to endothelial and neuronal cells. CM: conditioned medium, DM: diabetic condition, DM+Si: diabetic condition with silicon supplement, DM+Si+Fil: diabetic condition with silicon supplement, where a 0.2 µm filter was employed. Statistical analysis for B, E was performed using Student's *t‐*test, with significance defined as NS: not significant. Statistical analysis for H, J was performed using one‐way ANOVA, with significance defined as NS: not significant. All illustrations were created using Gallery software and are available for public utilization.

To investigate the form of substances that macrophages use for intercellular communication, a 0.2 µm filter (Fil) was used to remove large microvesicles from macrophage‐conditioned media. This approach aimed to examine the role of microvesicles in intercellular communication (**Figure** **4**A). In the DM+Si+Fil group, the levels of MMP and ATP in endothelial cells and neuronal cells were comparable to those in the DM group (Figure 4B; Figure S6, Supporting Information). Additionally, the differentiation of endothelial cells and neuronal cells in the DM+Si+Fil group did not significantly differ from that observed in the DM group (Figure 4B; Figure S6, Supporting Information). These results suggest that microvesicles, rather than cytokines, are involved in intercellular communication, which is essential for enhancing the energy metabolism and differentiation of endothelial and neuronal cells under a simulated diabetic condition.

To identify active substances in microvesicles, microvesicles in a macrophage‐conditioned medium were isolated by gradient centrifugation (Figure S7A,B, Supporting Information). Transmission electron microscopy (TEM) revealed the presence of extracellular mitochondria in the DM and DM+Si groups, but not in the control group (Figure 4C). Analysis of mitoTracker labeling, citrate synthase activity, and TOM20 protein level demonstrated that comparable mitochondrial content in microvesicles from the DM and DM+Si groups (Figure 4D; Figure S7C,D, Supporting Information). Furthermore, cell membranes containing mitochondria were observed in macrophages from the DM and DM+Si groups, indicating the release of mitochondria via microvesicles (Figure S8, Supporting Information). Besides, the TEM images of extracellular mitochondria in the DM and DM+Si groups also showed that the DM condition induced structural defects in extracellular mitochondria, such as cristae disarray and outer membrane swelling. These features were absent in the presence of silicon (DM+Si group). Furthermore, microvesicles in the DM+Si group were proved to contain more functional mitochondria, as evidenced by significantly elevated levels of respiratory chain complexes, MMP, and ATP. (Figure 4E) These observations indicate that a simulated diabetic condition led to mitochondria release from macrophages via microvesicles, both with and without silicon, which was discovered for the first time.^[^ ^31^ ^,^ ^32^ ^]^ At the same time, silicon did not affect the number of extracellular mitochondria but improved their quality.

To determine whether macrophage‐derived mitochondria are transferred to endothelial cells and neuronal cells, we conducted both in vitro and in vivo experiments. The efficacy of mitochondrial transfer was confirmed using an indirect co‐culture model with a conditioned medium (Figure 4F). Exogenous mitochondria (green, from macrophages) and endogenous mitochondria (red) were detected in vascular and neuronal cells. This finding is indicative of successful transfer under simulated diabetic conditions (Figure 4G). Quantitative analysis revealed a comparable proportion of exogenous to endogenous mitochondrial signals, regardless of silicon supplementation (Figure 4H). Filtration through a 0.2 µm filter resulted in the loss of green‐labeled mitochondria in vascular and neuronal cells, suggesting that mitochondrial transfer may also involve microvesicle‐mediated transport (Figure 4G,H). In a direct co‐culture model, results further confirmed the unidirectional transfer of mitochondria from macrophages to vascular and neuronal cells under simulated diabetic conditions (Figure S9, Supporting Information).

In order to investigate mitochondrial transfer during bone repair, macrophage‐depleted mice implanted with CS and SCS were injected with MitoTracker‐labeled macrophages (red) in the defect area. Results showed that macrophage‐derived mitochondria transferred to blood vessels and nerve fibers in diabetic mice (Figure 4I). MitoTracker levels in blood vessels and nerve fibers were similar in diabetic mice treated with either SCS or CS (Figure 4J). In contrast, immunofluorescence staining in healthy mice showed little co‐labeling. These findings suggest that mitochondrial transfer from macrophages to endothelial cells and neuronal cells under diabetic conditions through microvesicles.

Overall, these findings suggest that macrophages communicate with vascular cells and neuronal cells through mitochondrial transfer via microvesicles under diabetic conditions. Silicon does not affect the number of extracellular mitochondria but stimulates macrophages to produce functional mitochondria to facilitate vascularization and innervation via mitochondrial transfer (Figure 4K). Therefore, exploring the mechanisms by which silicon generates functional mitochondria in macrophages is crucial for understanding its role in diabetic bone repair.

### 2.5 Silicon Promotes Functional Mitochondrial Production by Altering Mitochondrial Fission via Mff Overexpression and Drp1‐Mff Pathway

**Figure 5:**
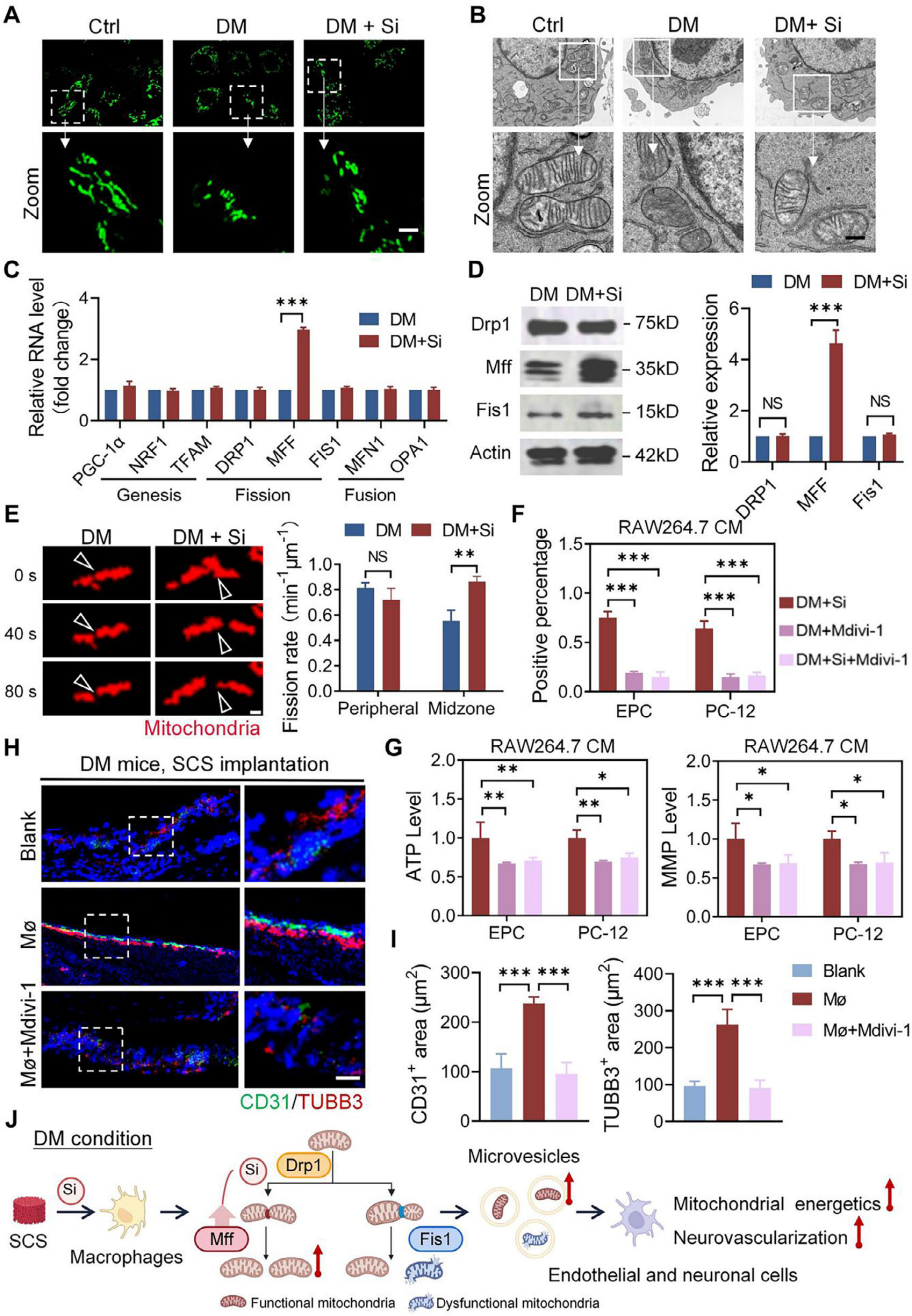
Silicon promotes functional mitochondrial production by altering mitochondrial fission under simulated diabetic (DM) conditions. A) Representative images of mitochondrial morphology in RAW264.7 cells under different culture conditions. Scale bar: 1 µm. B) Representative TEM images of mitochondrial morphology in RAW264.7 cells under different culture conditions. Scale bar: 100 nm. C) Quantitative analysis of gene expression levels related to mitochondrial biogenesis, fusion, and fission in RAW264.7 cells under DM or DM+Si conditions (*n *= 3). D) Analysis of mitochondrial fission protein expression in RAW264.7 cells under DM or DM+Si conditions (*n* = 3). E) Representative images and quantification of mitochondrial fission of macrophages under different culture conditions. Peripheral mitochondrial fission (less than 25% from a tip) and midzone fission (within the central 50%) were analyzed. Scale bar: 50 nm. F) Mitochondrial transfer from RAW264.7 to EPC and PC‐12 cells in different treatment groups were analyzed with flow cytometry (*n* = 3). G) Mitochondria function in EPC and PC‐12 cells in different treatment groups were analyzed by MMP and ATP levels (*n* = 6). H,I) Representative stained images and quantification of CD31^+^ vessels and TUBB3^+^ nerve within the calvarial defect site in different treatment groups (*n* = 4). Scale bar: 20 µm. J) Schematic showing silicon altering mitochondrial fission in macrophages to produce functional mitochondria. CM: conditioned medium, DM: diabetic condition, DM+Si: diabetic condition with silicon supplement, Mø: macrophages. Statistical analysis for C, D, E was performed using Student's *t‐t*est, with significance defined as **P < 0.01, ***P < 0.001, NS: not significant. Statistical analysis for F, G, I was performed using one‐way ANOVA, with significance defined as *P < 0.05, **P < 0.01, ***P < 0.001, NS: not significant. All illustrations were created using Gallery software and are available for public utilization.

To investigate the mechanism by which silicon participates in functional mitochondrial production, mitochondria in macrophages were analyzed carefully and comparatively. Mitochondrial motion tracks revealed that most mitochondria in the DM and DM+Si groups exhibited Brownian‐like random motion, while those in the control group were rarely motile (Figure S10, Supporting Information). Mitochondrial morphology analysis revealed that the length of mitochondria in macrophages derived from the DM and DM+Si groups was significantly shorter than those in the control group (**Figure** **5**A). Transmission electron microscopy further confirmed that the mitochondria in both the DM and DM+Si groups were shorter than those in the control group, with the DM group displaying notable deformation and swelling compared to the control and DM+Si groups (Figure 5B). These results indicate that mitochondrial fission is more active in diabetic conditions, and the activity of divided mitochondria after silicon supplementation is improved.

Mitochondrial morphology is regulated by the mitochondrial quality control system, which includes mitochondrial genesis, fission, and fusion.^[^ ^33^ ^]^ No significant differences in gene expression related to mitochondrial biogenesis and fusion were found between the DM and DM+Si groups (Figure 5C). However, a significant increase in the expression of mitochondrial fission factor (Mff) was observed in the DM+Si group (Figure 5C,D). Mitochondrial fission occurs through two mechanisms: mitochondrial fission 1 (Fis1)‐mediated fission at the periphery, leading to the formation of damaged and smaller mitochondria, and Mff‐mediated fission at the midzone, resulting in the proliferation of mitochondria.^[^ ^34^ ^]^ It was consistently observed that mitochondria in the DM group divided more frequently in the peripheral region, while those in the DM+Si group divided more frequently in the central region (Figure 5E) These results indicate that silicon enhances the expression of Mff, thereby influencing mitochondrial fission dynamics by promoting Mff‐mediated fission at the midzone.

The initiation of mitochondrial fission requires Mff to recruit dynamin‐related protein 1 (Drp1) to the mitochondrial outer membrane. To assess whether adequate mitochondrial fission is essential for neurovascularization via mitochondrial transfer, macrophages were treated with Mdivi‐1, a Drp1‐specific inhibitor, in both in vitro and in vivo diabetic conditions. Compared to silicon treatment, the addition of Mdivi‐1 significantly inhibited the ability of macrophages to transfer mitochondria (Figure 5F; Figure S11, Supporting Information). Notably, treatment with both Mdivi‐1 and silicon did not restore the mitochondrial transfer ability. Intracellular ATP and MMP levels indicated that neither the Mdivi‐1 treatment nor the combination of Mdivi‐1 and silicon significantly improved the mitochondrial function of vascular and neuronal cells compared to silicon treatment (Figure 5G). To further explore the in situ relevance of these findings, we examined the effect of macrophages on bone microenvironment reconstruction in diabetic mice implanted with SCS (Figure 5H,I). Diabetic mice transplanted with macrophages (*Mø*) exhibited more vascular and nerve regeneration than those not transplanted with macrophages. However, macrophages that underwent Mdivi‐1 pretreatment did not effectively promote vascular and nerve regeneration upon transplantation.

Overall, these results suggest that silicon regulates neurovascularization in diabetes through Mff overexpression and Drp1‐Mff‐mediated functional mitochondrial transfer (Figure 5J). This shift in fission dynamics promotes the proliferation of functional mitochondria within macrophages, which subsequently transfer to endothelial and neuronal cells, further enhancing energy metabolism and cell differentiation.

### 2.6 Silicon and P110 Combination Synergistically Promote Diabetic Bone Regeneration through Optimization of the Mitochondrial Fission Process

**Figure 6:**
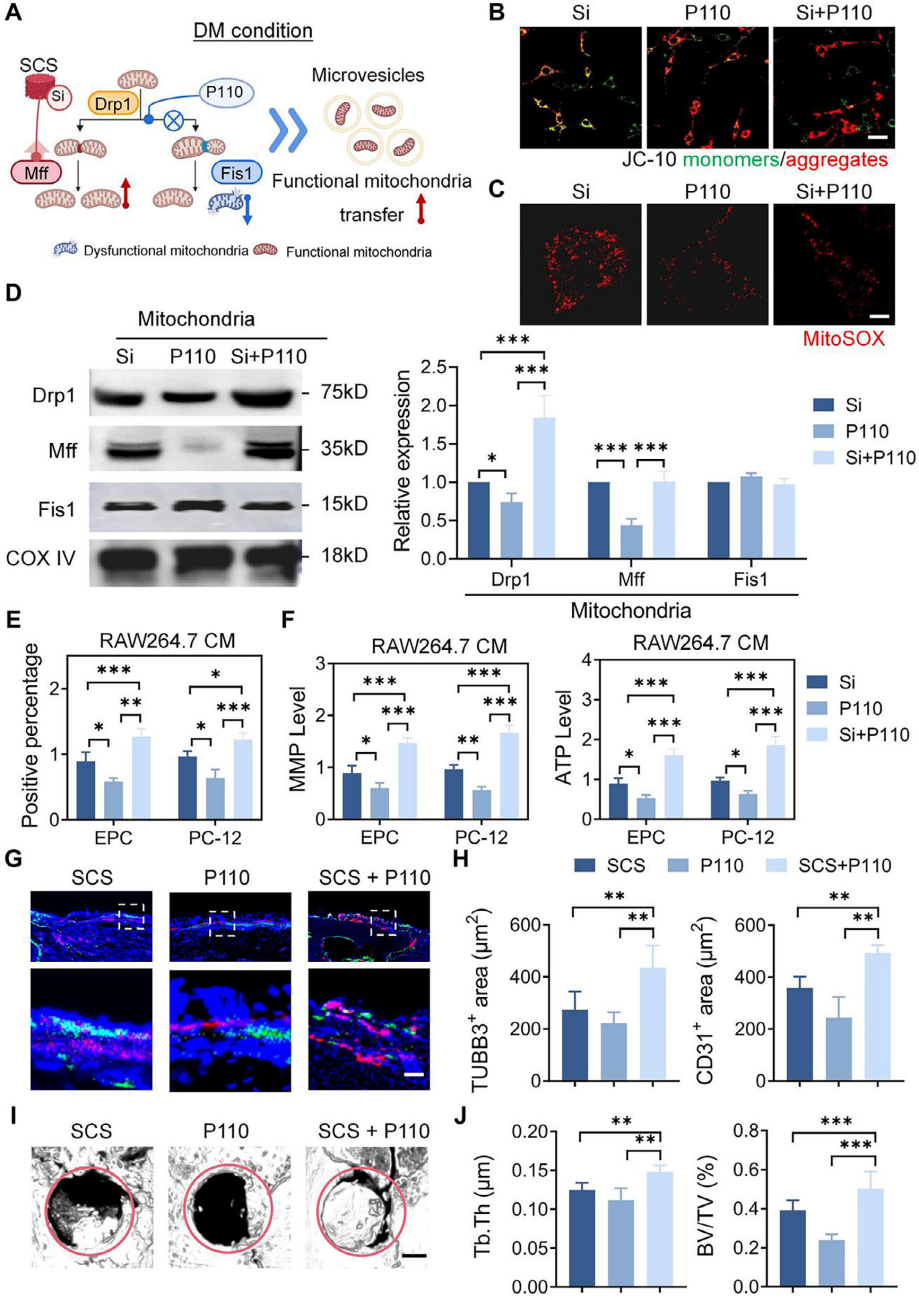
Combined Si and P110 therapy promotes diabetic bone regeneration. A) Schematic showing combination of Si and P110 altering mitochondrial fission to produce functional mitochondria. B) JC‐10 staining to determine mitochondrial membrane potential in RAW264.7 cells. Scale bar: 100 µm. C) Mitochondrion‐specific superoxide was detected by MitoSOX red staining in RAW264.7 cells. Scale bar: 10 µm. D) RAW264.7 cells were fractionated into membrane fractions, which were analyzed by Western blotting using designated antibodies (*n* = 3). E) Mitochondrial transfer from RAW264.7 to EPC and PC‐12 cells was analyzed in different groups using flow cytometry (*n* = 3). F) Mitochondria function in EPC and PC‐12 cells was analyzed in different groups by MMP and ATP levels (*n* = 6). G,H) Representative stained images and quantification of CD31^+^ vessels and TUBB3^+^ nerve within the calvarial defect site in different groups (*n* = 4). Scale bar: 20 µm. I,J) Representative micro‐CT images and quantitative analysis of newly formed bone in different groups (*n* = 4). Scale bar: 500 µm. CM: conditioned medium. Statistical analysis was performed using one‐way ANOVA, with significance defined as **P < 0.01, ***P < 0.001. All illustrations were created using Gallery software and are available for public utilization.

Although the above experiments have demonstrated that silicon‐based materials promote the repair of diabetic bone defects, silicon‐based materials did not achieve the desired bone repair effect during the experiment (Figure 1A), possibly because silicon primarily promotes physiological mitochondrial fission, while pathological mitochondrial fission still exists. To elucidate the specific mechanisms of silicon in diabetic bone defects, we examined the interactions of Drp1 with Fis1 and MFF under different conditions (Figure S12, Supporting Information). Immunoprecipitation experiments revealed that the simulated diabetic condition increased the expression of Fis1, while Silicon supplementation enhanced the expression of Mff. Importantly, neither the simulated diabetic condition nor Si supplementation affected the binding of Drp1 to Mff or Fis1. However, the combination of the simulated diabetic condition and Si supplementation promoted the interaction between Drp1 and Mff, while simultaneously reducing the interaction between Drp1 and Fis1. Based on this, diabetic bone defect repair could be further improved by reducing pathological mitochondrial fission. P110, a small molecule inhibitor, disrupts the interaction between Drp1 and Fis1, without affecting the binding of Drp1 to other mitochondrial receptors such as MFF.^[^ ^35^ ^]^ Thus, the combination of silicon and P110 could theoretically improve the efficacy of diabetic bone repair (**Figure** **6**A).

To assess the beneficial effects of P110 in conjunction with silicon on macrophage mitochondria under diabetic conditions, we examined mitochondria activity in macrophages. Mitochondrial activity in macrophages from the Si+P110 group was significantly increased compared to the Si group. This was demonstrated by a decrease in mitochondrial ROS levels (Figure 6B; Figure S13A, Supporting Information), and an increase in the ratio of JC‐10‐stained aggregates to monomers (Figure 6C; Figure S13B, Supporting Information). The macrophages from the Si+P110 group showed significantly reduced oxidative stress, as evidenced by increased activity of superoxide dismutase and catalase (Figure S13C, Supporting Information). Western blot analysis identified that there was no difference in Mff expression between the Si+P110 and Si groups in mitochondrial components. However, the expression of Drp1 was significantly increased in the Si+P110 group, indicating P110 promoted the recruitment of Drp1 in mitochondrial components (Figure 6D). These results suggest that P110 and silicon enhance mitochondrial activity through distinct fission patterns. To evaluate the effects of the silicon and P110 combination on endothelial and neuronal cells, we investigated mitochondrial transfer and cellular energy metabolism. The mitochondrial transfer rate in the Si+P110 combined group was higher than that in the P110 group or Si group (Figure 6E). Moreover, the Si+P110 group significantly increased MMP and ATP levels in endothelial cells and neuronal cells (Figure 6F). These experiments demonstrate the potential of combination therapy regimens to promote osteogenesis through mitochondrial transfer.

The effects of combination therapy on diabetic bone tissue regeneration were further investigated. In a diabetic cranial defect model, the expression levels of CD31^+^ blood vessels and TUBB3^+^ nerves in the SCS+P110 group were significantly higher than those in the SCS or P110 group (Figure 6G,H). Micro‐computed tomography results showed that bone defect repair in the SCS+P110 group was significantly improved, with the effect being significantly better than that of the SCS group or the P110 group (Figure 6I,J). Taken together, the combination of SCS and P110 produced a superior reparative effect on diabetic bone defects, including enhanced vascularization, innervation, and mineralization.

## 3 Discussion

Our results underscore the potential of a silicon‐based strategy to enhance the repair of diabetic bone defects. The mechanism of silicon involves promoting mitochondrial proliferation in macrophages through the upregulation of the Drp1‐Mff pathway, which facilitates the transfer of functional mitochondria to endothelial and neuronal cells. The integration of a silicified collagen scaffold with a Drp1‐Fis1 interaction inhibitor further optimizes the mitochondrial fission process, aiming to increase the availability of functional mitochondria and reduce the presence of pathological mitochondria. This approach demonstrates promising outcomes in promoting the regeneration of blood vessels, nerves, and mineralized tissue in diabetic models. These findings highlight the significance of targeting mitochondrial dynamics as a therapeutic strategy to enhance bone regeneration in individuals with metabolic disorders, further emphasizing the critical role of silicon in this process.

Intercellular communication is crucial for coordinating various cell types within a microenvironment. Recent studies have reported that mitochondria can move between cells or tissues through cell boundaries, a process known as horizontal transfer.^[^ ^36^ ^]^ In the context of tissue regeneration, mitochondrial transfer can have both positive and negative effects on cellular metabolism. Macrophages can protect endothelial cells from metabolic abnormalities by restoring oxidative phosphorylation through mitochondrial transfer,^[^ ^37^ ^]^ and similar protection can extend to neurons.^[^ ^38^ ^]^ However, the release of dysfunctional mitochondria by macrophages can induce pro‐inflammatory responses in endothelial cells^[^ ^39^ ^]^ or lead to inflammatory degeneration in neurons.^[^ ^40^ ^]^ In diabetes, there is an increase in mitochondria‐related proteins within circulating microvesicles, indicating elevated levels of circulating mitochondria.^[^ ^41^, ^42^ ^]^ In this study, we further elucidated the role of intercellular communication in the context of diabetic bone defects by highlighting the impact of silicon on mitochondrial transfer. Our findings demonstrate that silicon enhances the ability of macrophages to transfer functional mitochondria to endothelial and neuronal cells, thereby improving their metabolic function and regenerative capacity. This transfer occurs through microvesicles, which serve as conduits for intercellular communication under diabetic conditions. By promoting the exchange of healthy mitochondria, silicon not only mitigates the detrimental effects of diabetes on these critical cell types but also fosters a more conducive microenvironment for bone healing. Consequently, the facilitation of mitochondrial transfer represents a novel mechanism by which intercellular communication can be optimized to enhance tissue regeneration in diabetic bone defects.

Silicon is an essential micronutrient with potential regenerative effects mediated through mitochondrial regulation. It activates cellular defense mechanisms and, enhances resistance to ROS‐induced toxicity.^[^ ^27^, ^28^ ^]^ Although mitochondria are the primary site of ROS production, the effects of silicon on mitochondrial dynamics remain poorly understood. This study found that silicon independently upregulates Mff expression, thereby improving mitochondrial function in macrophages. Mitochondria fission factor is an important substrate of adenosine monophosphate‐activated protein kinase, a central metabolic sensor in various tissues.^[^ ^43^ ^]^ As the primary receptor for Drp1 in most mammalian cells, the Drp1‐Mff pathway plays a crucial role in regulating mitochondrial size,^[^ ^44^ ^]^ maintaining mitochondrial integrity,^[^ ^45^ ^]^ and promoting mitochondrial biogenesis.^[^ ^34^ ^]^ The findings demonstrate that silicon enhances Mff expression and facilitates Drp1‐Mff interactions, thereby promoting functional mitochondrial transfer from macrophages to endothelial and neuronal cells. This transfer is critical for improving cellular energy metabolism and supporting regenerative processes in diabetes where mitochondrial dysfunction is prevalent.

Mitochondrial fission plays a crucial role in the dynamics of the mitochondria lifecycle, facilitating the generation of new mitochondria and the removal of dysfunctional ones. Cellular stress triggers the binding of Drp1 to Fis1. This event causes mitochondrial fragmentation, ROS production, and metabolic collapse. P110 is an inhibitor designed to target the interaction between Drp1 and Fis1, mitigating pathological mitochondrial structure and functionality in various models, including neurodegeneration, ischemia, and sepsis.^[^ ^46^ ^]^ In this study, P110 was used to inhibit the activation of the Drp1‐Fis1 pathway and protect cells from mitochondrial damage in diabetic conditions. Our research also explored the role of silicon alongside P110. This combined approach demonstrated significant benefits in regulating mitochondrial function and promoting functional mitochondrial transfer. The silicon‐P110 combination resulted in notable improvements in diabetic bone defect repair, including vascularization, innervation, and mineralization. Therefore, a silicon‐based strategy represents a promising therapeutic strategy for enhancing hard tissue regeneration in diabetic conditions.

To our knowledge, this is the first study to elucidate the relationship between mitochondrial fission and mitochondrial transfer under diabetic conditions. Our findings suggest that the mitochondrial transfer process is closely linked to mitochondrial fission regulated by Drp1 in this context. Mitochondrial division is a complex process that involves multiple steps both upstream and downstream of Drp1 recruitment, beginning with the proper assembly of Drp1 at the mitochondrial membrane and progressing through Drp1‐driven membrane contraction to eventual membrane rupture. Drp1 adaptor proteins have shown that different adaptors exert distinct effects on mitochondrial fission and function, leading to varying expression patterns of Drp1 in specific cellular functions. Increased Drp1 activity and mitochondrial fission induced by diabetic conditions have been reported in the literature^.[^ ^46^, ^47^ ^]^ The role of changes in Drp1 activity in mitochondrial transfer has also been gradually recognized. Changes in Drp1 are reported to play an important role in mitochondrial transfer. Inhibition of the Drp1 activity following ischemia‐reperfusion reduces mitochondrial damage in astrocytes and facilitates the release of healthy mitochondria for subsequent transfer to neurons.^[^ ^48^ ^]^ The therapeutic effects of mitochondrial transfer from mesenchymal cells are believed to be regulated by Drp1 levels.^[^ ^49^ ^]^ Interestingly, recipient cells during mitochondrial transfer can exhibit either elevated or decreased levels of Drp1 phosphorylation.^[^ ^50^, ^51^ ^]^ Therefore, understanding the specific role of the Drp1 pathway provides valuable insights into how targeting mitochondrial dynamics could serve as a therapeutic strategy for individuals with metabolic disorders.

The recruitment of Drp1 to mitochondria during fission is mediated by multiple receptors including Fis1 and Mff. Under simulated diabetic conditions, Drp1 exhibits increased binding with Fis1, potentially disrupting mitochondrial dynamics by favoring dysfunctional fission pathways. However, silicon supplementation significantly shifts Drp1 interactions by increasing its binding to Mff, while reducing its association with Fis1. These findings suggest that silicon promotes functional mitochondrial fission by enhancing Drp1‐Mff recruitment to restore mitochondrial homeostasis. The competitive interaction between Mff and Fis1 has been previously reported. Increased Fis1 expression is associated with decreased Mff expression,^[^ ^52^ ^]^ while Fis1 knockdown results in Mff upregulation.^[^ ^53^ ^]^ Notably, targeted inhibition of the Drp1‐Fis1 pathway has demonstrated significant anti‐tumor effects, whereas Drp1‐Mff inhibition has been linked to pro‐tumor activity.^[^ ^54^ ^]^ These observations suggest that fission‐dependent mitochondrial regulation in diabetes directly influences mitochondrial homeostasis and macrophage‐mediated mitochondrial transfer, which propagates regenerative signals. The incorporation of silicon and P110 in the therapeutic strategy not only protects mitochondria but also modulates the balance between Fis1 and Mff to optimize mitochondrial function.

### 3.1 Limitations and Future Directions

This study has several limitations that should be acknowledged. First, we used a streptozotocin (STZ)‐induced diabetes model, primarily reflecting type 1 diabetes, which may not fully represent the complexities of type 2 diabetes. Future studies should consider models that better mimic the pathophysiology of type 2 diabetes. Second, the cell lines used, such as RAW264.7, EPC, and PC‐12 cell lines, may not accurately reflect the behavior of primary cells in vivo. Responses observed in these cell lines could differ from those in primary cells or in a more complex tissue environment. Last, various external factors, including the local microenvironment and interactions with other cell types, could influence our results. Acknowledging these limitations is important for understanding the implications of our findings and for guiding future research.

Regarding the translational potential of silicon, bioavailability and safety are essential considerations. Silicon is primarily obtained from dietary sources, and its absorption can be influenced by gut health and nutritional status. Existing literature indicates that silicon supplementation is generally safe, making it a viable candidate for clinical application. The use of silicon supplements, such as silicified collagen scaffolds, shows promise for enhancing hard tissue regeneration in diabetic conditions. Our findings demonstrate that silicon improves vascularization and innervation by facilitating the transfer of functional mitochondria from macrophages to endothelial and neuronal cells. To promote the clinical application of silicified collagen scaffolds in treating diabetic bone defects, further preclinical experiments in large animals are needed in the future.

The synergistic effect of silicon and P110 represents a promising therapeutic strategy for promoting hard tissue regeneration in patients with diabetes; however, certain limitations currently exist. It is essential to explore various delivery routes for P110, such as nanoparticles or biodegradable stents, to achieve increased local drug concentrations while minimizing systemic exposure. Additionally, it is important to recognize that the mechanical strength of silicon‐based materials may be lower than that of some other biological materials, such as hydroxyapatite or polylactic acid, which could limit their application in load‐bearing sites. Overall, a comprehensive understanding of the advantages and limitations of silicon‐based materials, along with strategic modifications, may enhance the treatment of bone defects in diabetic patients.

## 4 Conclusion

Our findings reveal that silicon exerts strong protective effects in diabetic bone defect models by selectively promoting Drp1‐Mff activity. Silicon facilitates precise regulation of mitochondrial dynamics to enable functional mitochondrial transfer. The findings demonstrate the effectiveness of silicon in alleviating diabetic bone defects and its potential as an innovative therapeutic approach.

## 5 Experimental Section

### Preparation of SCS

Silicified collagen scaffolds were prepared using a previously‐reported protocol (Figure S1A, Supporting Information).^[^ ^29^ ^]^ A silicifying medium was prepared by mixing Silbond 40 (Evonik Industries, Essen, Germany), ethanol, deionized water, and 37% hydrochloric acid in a molar ratio of 1.875:396.79:12.03:0.0218. Reconstituted collagen scaffolds (Ace Surgical Supply, Brockton, MA, USA) with a diameter of 4 mm were first stirred and soaked in a 36 mm choline chloride solution and then soaked in an equal amount of silicifying medium for 7 days. After drying, the SCS was sterilized by cobalt‐60 irradiation for further biological experiments.

### Silicic Acid Release from SCS

Collagen scaffolds (100 mg, without silicification) or SCS (100 mg, before silicification) were soaked in 10 mL of Dulbecco's Modified Eagle Medium (DMEM, Gibco, Gaithersburg, MD, USA). The supernatant was collected at different time‐points for analysis. The release of silicic acid from SCS in the DMEM was measured using spectrophotometry and compared to the release from CS extract. After testing, the remaining SCS extracts were transferred to new culture bottles and diluted into Si supplement medium for subsequent cell culture experiments. To ensure consistency and stability in multiple experiments, all Si supplement media were stored for at least 24 h before use.

### Atomic Force Microscopy

Atomic force microscopy (AFM) was used to examine the microstructure and surface roughness of CS and SCS. The scaffolds were placed in plastic molds and embedded with optimal cutting temperate (OCT) compound. The embedded specimens and sliced into 40 µm thick sections. The slices were rinsed, dried on a cover slip, and examined using AFM. The surface morphology of the collagen fibrils was generated using the analysis software provided by AFM.

### Diabetic Mouse Model

Animal experiments were conducted with approval from the Institutional Animal Care and Use Committee of the Fourth Military Medical University and in accordance with established guidelines. The project was granted ethical approval, designated by the approval number 2022‐kg‐009. A schematic of the diabetic mouse model and the timeline for treatment is shown in Figure S2A (Supporting Information). To induce diabetes, each mouse was injected intraperitoneally with the β‐cell toxin streptozotocin (STZ, 100 mg dissolved in vehicle kg^−1^ body weight; Sloarbio, Beijing, China). The STZ‐injected mice were fed a commercial high‐fat diet (HFD, M10160, BioPike, Beijing, China) during the experimental phase. Blood glucose levels were measured from non‐fasted blood samples using a glucose monitor (Accusure 660, Yuwell, Jiangsu, China). Mice with median glucose levels greater than 17 mmol L^−1^ were included in the diabetic (DM) group. Body mass was also recorded for all mice at each time‐point during the experiment.

### Murine Cranial Defect Model

The bone regeneration potential of different treatment regimens was examined by creating a critical‐sized bone defect in the cranium of each mouse. Anesthesia was performed by intraperitoneal injection of sodium pentobarbital (40 mg k^−1^g body weight). After disinfection and anesthesia, a skin incision was made to expose the skull, and the periosteum was removed. A 4 mm diameter full‐thickness defect was created on the central parietal bone using a sterile trephine bur (Precision Tools, Shanghai, China).

### Murine Macrophage‐Depletion Model

Liposomes containing clodronate (available from clodronateliposomes.com) were used to deplete macrophages in vivo. Twenty microliters of the liposomes were delivered locally to the cranial defect area. A systemic dose of liposomes (10 µL g^−1^) was administered every 48 h. This treatment protocol commenced 48 h prior to surgery and continued until the day of euthanasia (Figure S5A, Supporting Information). The effect of macrophage depletion was determined by F4/80 immunofluorescence staining.

### Histology

Histological examination was used to evaluate the regeneration of the bone microenvironment. The calvaria of the animals were obtained immediately after euthanasia, fixed in 4% paraformaldehyde, demineralized in 10% EDTA for two weeks, and embedded in paraffin or OCT compound. Four micrometer‐thick sections were obtained for H&E staining (Servicebio, Wuhan, Hubei, China) and examined using a light microscope (BX61, Olympus, Tokyo, Japan). In addition, 15 µm‐thick sections were obtained for immunofluorescence staining. All sections were prepared in a plane parallel to the sagittal suture and through the center of the defect.

For immunofluorescence staining, the primary antibodies used were F4/80 antibody (ab6640, Abcam, Cambridge, UK), CD31 antibody (sc‐376764, Santa Cruz Biotechnology, Inc., Dallas, TX, USA), and beta‐III tubulin antibody (TUBB3, ab78078, Abcam). The corresponding secondary antibodies were Alexa Fluor antibodies (Jackson ImmunoResearch Laboratories, Inc., West Grove, PA, USA). Cell nuclei were stained with 4′,6‐diamidino‐2‐phenylindole (DAPI; ab228549; Abcam). The stained sections were imaged using confocal laser scanning microscopy (CLSM, FV1000, Olympus). Fluorescence intensities were quantified with the ImageJ software (National Institute of Health, Methasda, MD, USA).

### Micro‐Computed Tomography

Micro‐computed tomography scans (Siemens Preclinical, Knoxville, TN, USA) were performed at a resolution of 21 µm to examine bone regeneration in the murine cranial defects. The acquired images were reconstructed using the Inveon Research Workplace software (Siemens Medical Solutions USA, Inc., Hoffman Estates, IL, USA). A cylindrical region of interest (ROI) was positioned over the defect site. The bone volume to total volume ratio (BV/TV) and trabecular thickness (Tb.Th) in the ROI were calculated using the Inveon Research Acquisition software.

### Cell Culture

The cell lines used in the present study were RAW264.7 (macrophage; Cyagen Biosciences, Guangdong, China), endothelial progenitor cells (EPCs; Jennio Biotech, Guangdong, China), and pheochromocytoma cells (PC‐12, Jennio Biotech). The cells were initially cultured at 37 °C in a humidified atmosphere of 5% CO_2_. The culture medium consisted of DMEM with 1% antibiotic‐antimycotic, and 10% fetal bovine serum (Gibco, Gaithersburg, MD, USA).

Primary murine macrophages were directly isolated and purified. Briefly, PBS solution was injected into the mouse peritoneal cavity to promote sufficient contact of the wash solution. The collected peritoneal lavage fluid was centrifuged at 200 rpm for 5 min at 4 °C. An appropriate amount of red cell lysis buffer was added to the precipitate. Centrifuging was performed again at 1000 rpm for 5 min at 4 °C. The cells were re‐suspended and seeded into a well plate, and washed with PBS twice. The final adherent cells were the desired macrophages.

Primary murine endothelial cells were located on the luminal surface of blood vessels and could be isolated and cultured from mouse aortas. Briefly, a PBS solution containing 1000 U mL^−1^ heparin was injected into the left ventricle of the mouse for cardiac perfusion. The aorta segment was transplanted onto a matrix gel surface, ensuring that the lumen was facing downward. A DMEM complete medium was added to cover the aorta segment. For the first passage, the adherent endothelial cells were digested with neutral protease. The cell suspension was collected and centrifuged at 1000 rpm for 5 min at room temperature. The cells were re‐suspended in fresh culture medium and seeded onto a well plate coated with 0.1% gelatin.

Primary peripheral neurons might be produced by directly isolating mouse dorsal root ganglion cells, followed by enzymatic hydrolysis. After anesthesia, a mouse spine was dissected, and the dorsal root ganglion was visible as a transparent globular ganglion in the spinal recess. Bilateral dorsal root ganglia were then isolated one by one and immersed in a pre‐cooled DMEM medium. To digest the ganglion tissue, 0.1% collagenase and 0.25% trypsin were added, and the cell density was adjusted before seeding onto a well plate. The growth medium for dorsal root ganglion was neurobasal‐A medium supplemented with 0.5 mm L^−1^ glutamine, 20 ng mL^−1^ nerve growth factor, 2% B‐27, 100 U mL^−1^ penicillin, and 100 µg mL^−1^ streptomycin.

### Preparation and Characteristics of a Simulated Diabetic Culture Condition

To simulate an in vitro diabetic condition, the cells were treated with an additional 25 mm glucose and 1 mm H_2_O_2_ for 12 h. The effect of a simulated diabetic condition on different types of cells was measured by cell viability and intracellular ROS accumulation. Cell viability was determined using a CCK‐8 assay kit, while intracellular ROS accumulation was detected by a DCFH‐DA assay kit (Beyotime, Shanghai, China). Quantitative evaluation was performed according to the manufacturer's instructions.

### Cell Metabolism

Oxygen phosphorylation and glycolysis were measured to evaluate the effects of silicon on energy metabolism in a simulated diabetic condition. The experiment was performed using a bioenergetic assay (XFe96; Seahorse Bioscience, Billerica, MA, USA). Macrophages, endothelial cells, and neuronal cells were inoculated into Seahorse XF96 microplates with at least four duplicate wells for each cell type. The cells were treated according to a preset experimental protocol for ≈24 h. The oxygen consumption rate and extracellular acidification rate were measured to understand the aerobic phosphorylation metabolism of mitochondria, as well as the metabolic activity of the cells to glucose and their glycolysis capacity.

### Mitochondrial Activity

The MMP assay kit with a JC‐10 fluorescent probe (Solarbio) was used to measure MMP. The relative levels of fluorescence were measured using flow cytometry (BD Biosciences, Franklin Lakes, NJ, USA) or CLSM. The cationic dye JC‐10 exhibited two different fluorescence colors depending on the MMP. When MMP was high, JC‐10 formed aggregates that emitted red fluorescence. When MMP was low, JC‐10 remained in its monomeric form, which emitted green fluorescence. The ratio of JC‐10 red/green fluorescence intensity was used to estimate the MMP, according to the manufacturer's protocol.

Adenosine triphosphate (ATP) levels were measured using the colorimetric ATP assay kit (Solarbio). Cells to be tested were trypsinized and centrifuged. The supernatants and ATP standard solutions were mixed with dilution buffer provided with the ATP assay kit. The ATP level was measured using a microplate reader (BioTek Instruments Inc., Winooski, VT, USA) at an absorbance of 340 nm.

Citrate synthase (CS) activity was measured according to the manufacturer's instructions (Solarbio). Acetyl co‐enzyme A, oxalacetic acid, and 5,5′‐dithiobis(2‐nitrobenzoic acid) were combined in Tris·HCl buffer. The rate of 5‐thio‐2‐nitrobenzoic acid formation was measured at 30 °C using a plate reader. Citrate synthase activities were normalized to protein concentration/sample.

### Mitochondria Staining

MitoTracker Green or MitoTracker deep red (ThermoFisher Scientific, Waltham, MA, USA) was used to label intracellular and extracellular mitochondria. Cells were washed with serum‐free medium and incubated with 200 nm MitoTracker for 30 min. The stained cells were examined with CLSM and images were analyzed using the Mitochondrial Network Analysis software (MiNA 3.0.1) to quantify the mitochondrial network parameter values.

For live cell observation, the cells were seeded on glass coverslips and labeled with MitoTracker Green or MitoTracker deep red. The stained cells were observed and photographed in the live cell workstation. For fixed cell or tissue observation, a MitoTracker deep red probe was used and samples were fixed in 4% paraformaldehyde, incubated with 0.3% Triton X‐100 and 1% pre‐immune goat serum. To observe the position of marked mitochondria in relation to other markers, the samples were incubated with the specified primary antibody and the appropriate secondary antibody. The samples were mounted in an anti‐fade mountant and stained with DAPI for CLSM examination.

### Tube Formation

Matrigel matrix (Corning Life Sciences, Lowell, MA, USA) was used to evaluate the effect of conditioned medium on tube formation from EPCs. The gel matrix was applied to well plates and incubated at 37 °C for 30 min prior to cell seeding. The EPCs were seeded on the polymerized Matrigel and incubated in different conditioned media according to the group designation, at 37 °C for 6 h. Tube lengths were examined using light microscopy. Lengths were determined from 3 different wells in 6 independent replicates, using the ImageJ software.

### Neurite Length

Nerve growth factor (50 ng mL^−1^; MilliporeSigma) was added to conditioned media to evaluate the effect of conditioned medium on PC‐12 differentiation, The average length of neurites was evaluated based on microscopic images. Lengths were determined for 20 cells in 6 independent replicates using the ImageJ software. Neurites with lengths that were at least twice as long as the body length of the cell were measured.

### In Vitro Mitochondrial Transfer

To observe mitochondrial transfer, RAW264.7, EPC, and PC‐12 cells were first incubated separately for 30 min in a staining solution containing a MitoTracker probe. After washing with PBS three times to remove the free probe, the RAW264.7 cells were co‐cultured with PC‐12 or EPC in different incubation environments (Figure 4F).

In the indirect co‐culture model, PC‐12 or EPC were placed in macrophage‐conditioned media with different treatments. The RAW264.7 mitochondria in EPC/PC‐12 cells were observed by CLSM. The mitochondrial transfer rate was quantitatively measured by CLSM (50 cells in 6 independent replicates) or flow cytometry (3 different wells in 6 independent replicates).

In the direct co‐culture models, endogenous or exogenous mitochondrial distribution in EPC or PC‐12 cells was observed by CLSM. Under all conditions, the initial inoculation density of the three cells was the same, and the co‐culture time was not less than 12 h.

### In Vivo Mitochondrial Transfer

RAW264.7 macrophages were labeled with MitoTracker Red and were intravenously administered through the mouse tail vein (3 × 10^5^ cells mouse^−1^). The mice were euthanized at designated time‐points. The cranium was harvested and mitochondria distribution was visualized by CLSM. Analysis and quantification were done using ImageJ software.

### Particle Size

For microvesicle collection from the medium, the supernatant was collected and centrifuged at 2000 g for 10 min and 10 000 g for 30 min (Figure S7A, Supporting Information). Prior to analyzing particle size distribution, the samples were diluted in PBS to obtain a range of 60% transmittance. Analysis was conducted using the NanoSight NTA Software (Litesizer 500, Anton Paar, GmbH, Graz, Austria).

### Transmission Electron Microscopy

The supernatant was centrifuged for visualization of extracellular vesicles. A small amount of the vesicle pellet was placed on a carbon‐coated copper grid, and stained with 2% uranyl acetate. Excess stain was removed, and the grid was allowed to dry before examination using a transmission electron microscope (TEM).

For ultrastructural analysis, the treated cells or vesicle pellets were fixed with 3% glutaraldehyde and then post‐fixed with 1% osmium tetroxide. The fixed specimens were dehydrated in an ascending ethanol series (50‐100%), infiltrated, and embedded in epoxy resin. Seventy nanometer‐thick sections were prepared and stained with uranyl acetate and lead citrate. The stained sections were examined with a JEM‐1400 TEM (JEOL, Tokyo, Japan) at 120 kV.

### Standard Fluorescence‐Activated Cell Sorting

Standard fluorescence‐activated cell sorting (FACS) was performed using the BD LSR II or BD Fortessa, as described previously. To sort extracellular mitochondria in a conditioned medium, the supernatant was collected from RAW264.7 cells labeled with MitoTracker (Figure S7A, Supporting Information). To sort exogenous mitochondria in EPC and PC‐12 cells, these cells were co‐cultured indirectly with the MitoTracker‐labeled RAW264.7 cells (Figure 4F). Cell sorting was performed using an unstained or phenotype control for determining appropriate gates, voltages, and compensations required in multivariate flow cytometry.

### Real‐Time Quantitative Polymerase Chain Reaction

Gene expressions were evaluated using real‐time polymerase chain reaction to investigate the relationship between silicon stimulation and mitochondrial dynamics. The following genes were examined: PGC‐1α, NRF1, TFAM, MFN1, OPA1, DRP1, FIS1, and MFF. Sense and antisense primers of these genes were designed based on published cDNA sequences (Table S1, Supporting Information). Total RNA was isolated using Trizol reagent (Invitrogen, ThermoFisher Scientific). Complementary DNA was synthesized using the PrimeScript RT reagent kit (Takara Bio Inc., Shiga, Japan). The RT‐PCR experiment was performed using a 7500 Real Time PCR System (Applied Biosystems, Carlsbad, CA, USA). Gene expression was normalized to glyceraldehyde 3‐phosphate dehydrogenase (GAPDH). Results obtained after calibration with the GAPDH expression level were calculated using the 2^^−ΔΔCt^ method and presented as fold increased relative to the non‐stimulated control.

### Western Blot

Cellular proteins were extracted and quantified using a BCA assay kit (Beyo`time, P0012S). Equal amounts of proteins were dissolved in SDS‐PAGE buffer, separated by electrophoresis, and transferred to polyvinylidene difluoride membranes. After blocking, the proteins were incubated with rabbit anti‐Fis1 (10956‐1‐AP, Proteintech, Rosemont, IL, USA), rabbit anti‐Mff (17090‐1‐AP, Proteintech), and rabbit anti‐p‐Drp1 (12957‐1‐AP, Proteintech) at 4 °C overnight, followed by incubation with a goat anti‐rabbit IgG (Yeasen, Shanghai, China) secondary antibody. β‐tubulin (#2146, Cell Signaling, Portland, OR, USA) and COX IV (#4844, Cell Signaling) were used as the internal control. Stained bands were visualized with the chemiluminescence detection system. The gray values were analyzed using Image‐J software.

### Co‐Immunoprecipitation

Immunoprecipitation Kit with Protein A+G Magnetic Beads (Beyotime, P2179S) was employed. The pre‐treated cells were first lysed with lysate, the antibodies were then incubated with magnetic beads for 30 to 60 min, the lysate was then incubated with the coupled magnetic beads for 2 h, and a single antibody was added. Finally, the magnetic beads were eluted with eluent to obtain the target protein. Electrophoresis using SDS‐PAGE was used to evaluate the related proteins.

### Cellular Resistance to Oxidative Stress

Lipid peroxidation was analyzed using malondialdehyde (MDA) levels. Antioxidant activity was analyzed using glutathione (GSH) content. The treated cells were lysed and centrifuged, and the resulting supernatants were collected for analysis, following the manufacturer's instructions.

### Mitochondrial Fission Inhibitor

The effects of inhibiting Drp1 were examined using two inhibitors: Mdivi‐1 and P110. The Mdivi‐1 (MilliporeSigma) was used at 30 µm. RAW 264.7 cells were pretreated with Mdivi‐1 for 30 min prior to further experiments. P110 was synthesized by QYAOBIO Biotechnologies (Shanghai, China). The purity of the peptides used was greater than 95%, as measured by reverse phase‐high pressure liquid chromatography. RAW 264.7 cells were treated with P110 at 1 µm. Depending on the specific experiment, the cells were examined 24 h after incubation with the P110 inhibitor. In addition, P110 was also administered to diabetic mice at a dose of 0.5 mg k^−1 ^g day^−1^. The P110 inhibitor was dissolved in 0.2 mL of saline and injected intraperitoneally.

### Statistical Analysis

All surgical procedures, as well as quantitative histological and behavioral analyses, were conducted in a blinded manner. Data are presented as means ± standard deviations (SD). Prior to statistical analysis, the normality of the data distribution was assessed using the Shapiro–Wilk test, and homoscedasticity was evaluated using Levene's test. For comparisons between two groups, the Student's *t‐*test was employed, utilizing a two‐sided testing approach with a significance level set at *α* = 0.05. For comparisons involving more than two groups, one‐way analysis of variance (ANOVA) was conducted, followed by post‐hoc Tukey's pairwise comparison procedures to identify specific group differences. Sample sizes (*n*) for each analysis are provided in the respective figure legends and results sections. All statistical analyses were performed using the GraphPad Prism 5 software (GraphPad Software, La Jolla, CA, USA).

## Conflict of Interest

The authors declare no conflict of interest.

## Supporting information



Supporting Information

## Data Availability

The data that support the findings of this study are available from the corresponding author upon reasonable request.
