# Supplementary material for: Silicon Enhances Functional Mitochondrial Transfer to Improve Neurovascularization in Diabetic Bone Regeneration
Source: Adv Sci (Weinh). 2025 Mar 24;12(19):2415459. doi: 10.1002/advs.202415459 (PMC12097102; doi:10.1002/advs.202415459)
Supplement: Supplementary file 1 — Supporting Information [file ADVS-12-2415459-s001.docx]

**Supplementary Information**

**Silicon improves diabetic bone repair through mitochondrial transfer**

**Table of Contents**

*Yu-Xuan Ma^1#^, Chen Lei^1#^, Tao Ye^1#^, Qian-Qian Wan^1^, Kai-yan Wang^1^, Yi-na Zhu^1^, Ling Li^1^, Xu-fang Liu^1^, Long-zhang Niu^1^, Franklin R. Tay^2^, Zhao Mu^3^*, Kai Jiao^4^* and Li-Na Niu^1^**

**SI-1.** Preparation and characteristics of silicified collagen scaffold.

**SI-2.** Preparation and characteristics of DM mice.

**SI-3.** Effect of silicified collagen scaffold on bone deposition in DM mice.

**SI-4.** Preparation and characteristics of DM condition medium.

**SI-5.** Preparation and characteristics of macrophage-depleted DM mice.

**SI-6.** Silicon collaborates with macrophages to enhance angiogenesis and nerve regeneration via microvesicles.

**SI-7.** Isolate and identify extracellular vesicles produced by macrophages under DM or DM+Si conditions.

**SI-8.** Macrophages expel mitochondria through extracellular vesicles under DM condition.

**SI-9.** Mitochondria released from macrophages transfer to endothelial and neuronal cells.

**SI-10.** DM condition changes the mitochondrial movement in macrophages.

**SI-11.** Effect of macrophage mitochondrial division on mitochondrial transfer under DM condition.

**SI-12.** Silicon enhanced the interaction between Mff and Drp1 under induced simulated diabetic condition.

**SI-13.** P110 reduces mitochondrial reactive oxygen species and improves oxidative stress in macrophages.

**SI-14.** Primer sequences used for RT-PCR in the present study.

**SI-1.** Preparation and characteristics of silicified collagen scaffold.

**
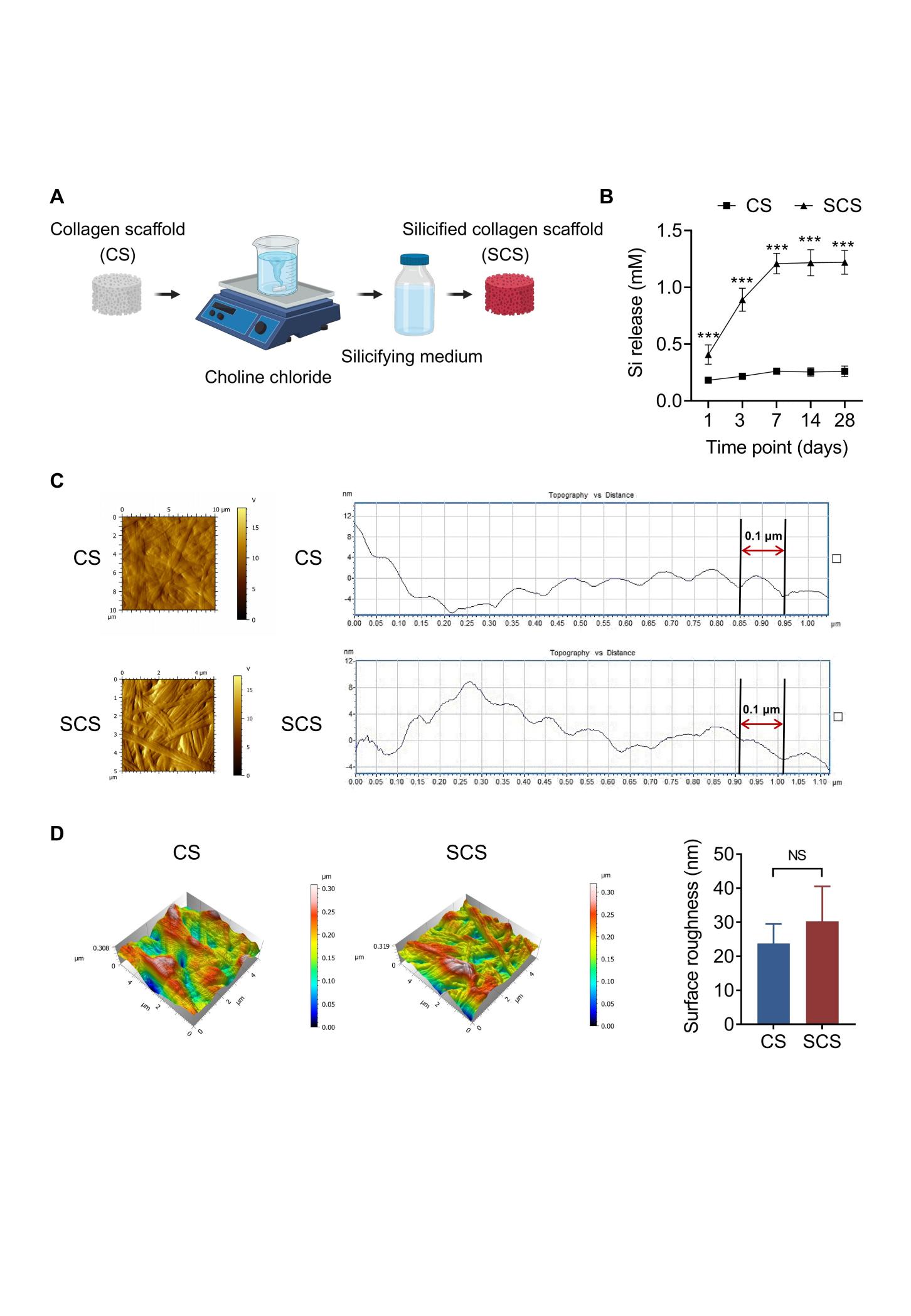
**

**Fig.SI-1 Preparation and characteristics of silicified collagen scaffolds.** A) Schematic diagrams of SCS preparation. B) Silicic acid release from CS and SCS at different time points (n = 4). C) Representative images of two-dimensional surface topography and periodic profiles of height measurement. D) Representative images and quantitative analysis of three-dimensional surface topography roughness (n = 4). ****P* < 0.001, NS: not significant.

**SI-2.** Preparation and characteristics of DM mice.

**
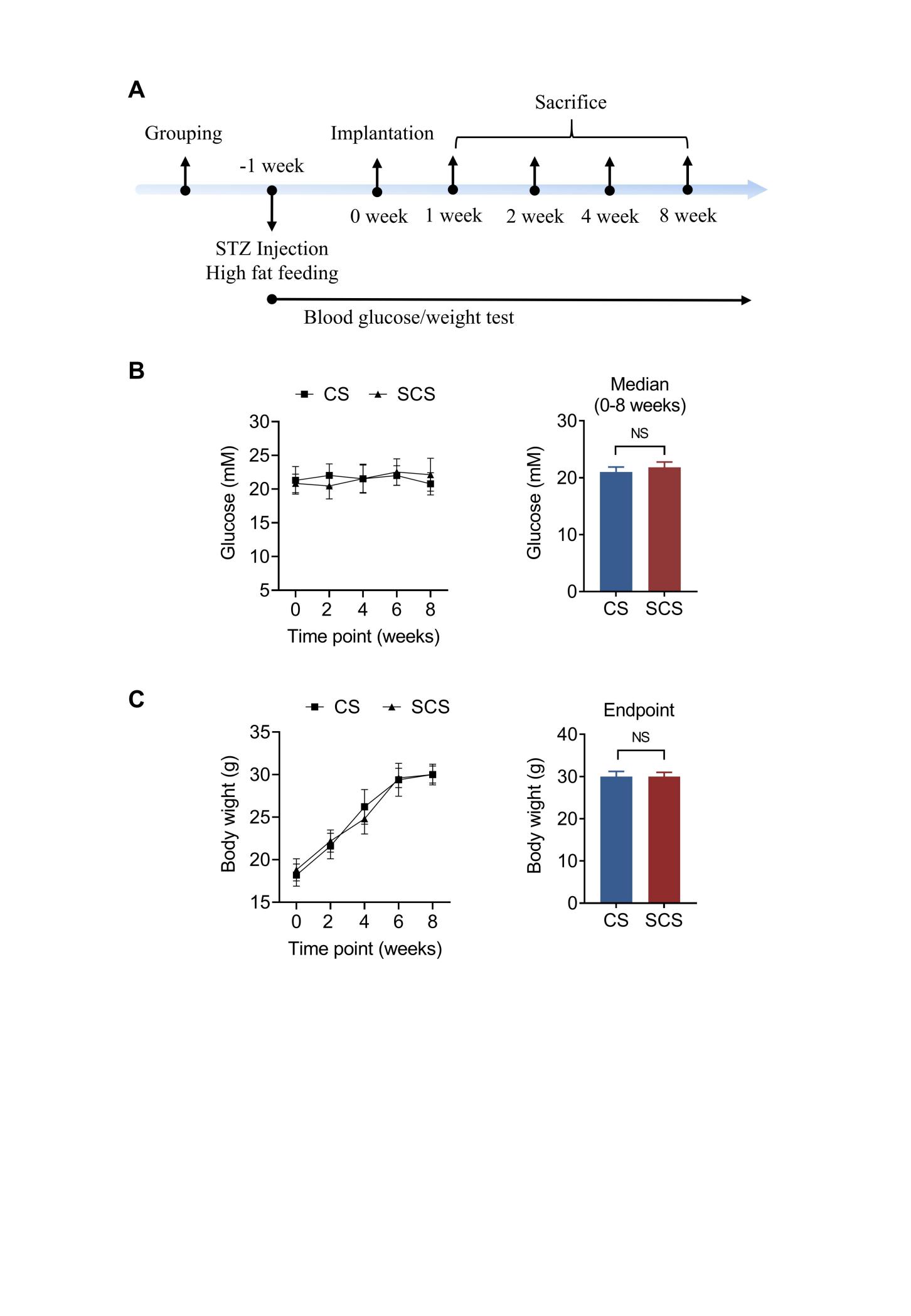
**

**Fig.SI-2 Preparation and characteristics of DM mice.** A) Schematic diagrams of DM Mice. B) Glucose level of DM mice with CS or SCS treatment at different time points (n = 4). C) Body wight of DM mice with CS or SCS treatment at different time points (n = 4). NS: not significant.

**SI-3.** Effect of silicified collagen scaffold on bone deposition in DM mice.


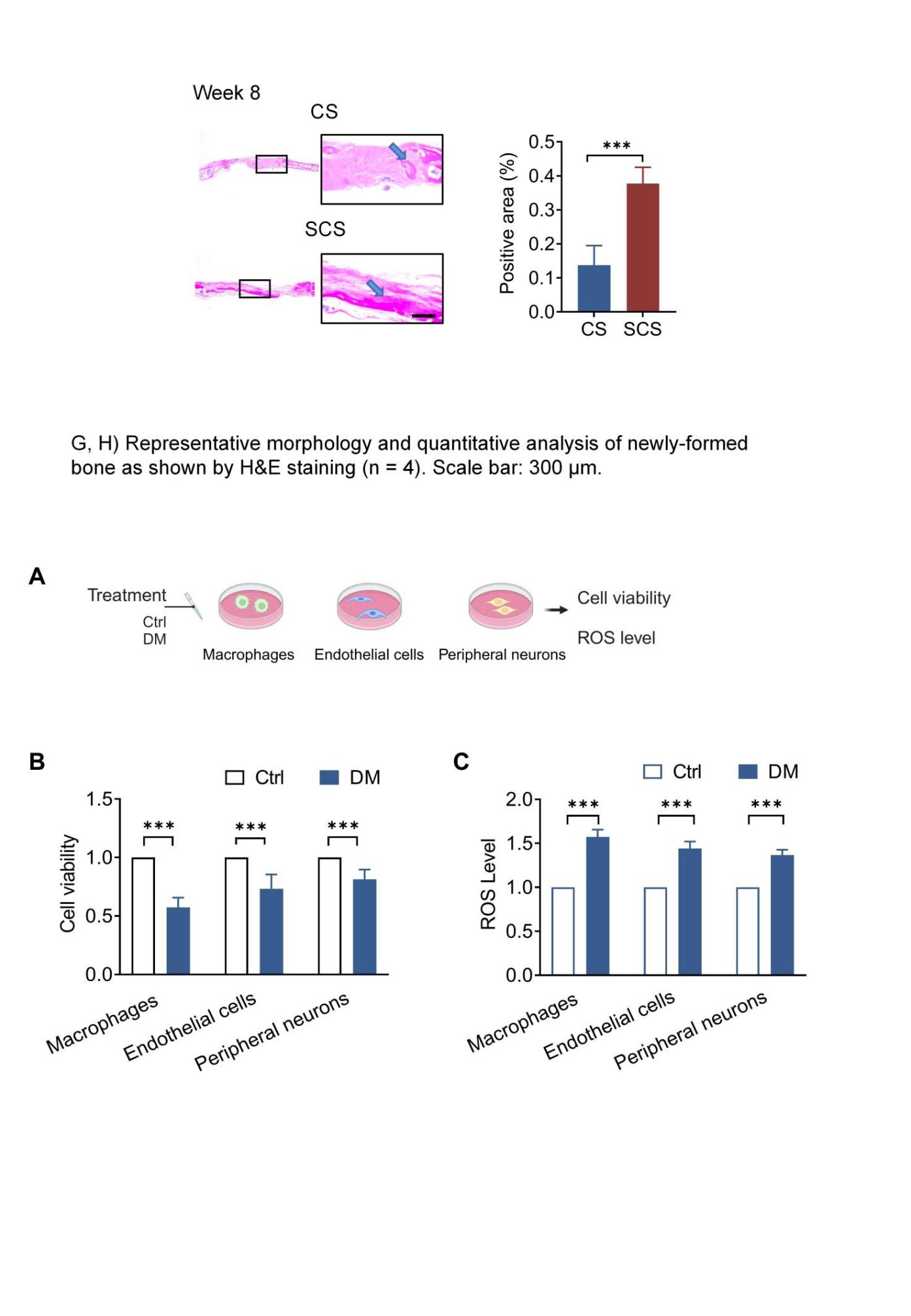


**Fig.SI-3 Effect of silicified collagen scaffold on bone deposition in DM mice.** Representative morphology and quantitative analysis of newly-formed bone as shown by H&E staining (n = 4). Scale bar: 300 μm.

**SI-4.** Preparation and characteristics of DM condition medium.

**
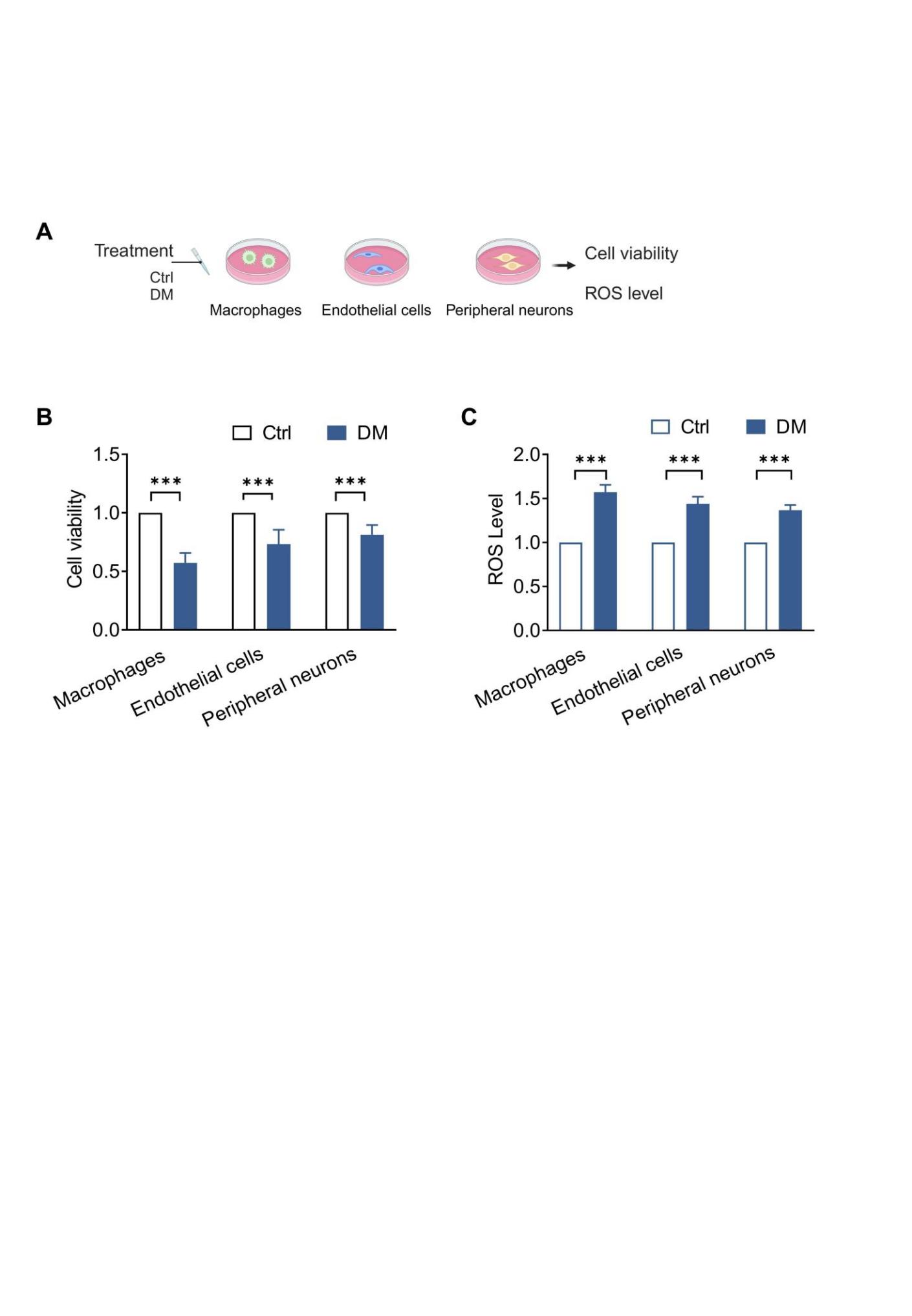
**

**Fig.SI-4 Preparation and characteristics of DM condition medium.** A) Schematic diagrams of DM condition preparation. B) Cell viability of various cell types under Ctrl or DM conditions (n = 6). C) ROS level of various cell types under Ctrl or DM conditions (n = 6). ****P* < 0.001.

**SI-5.** Preparation and characteristics of macrophage-depleted DM mice.


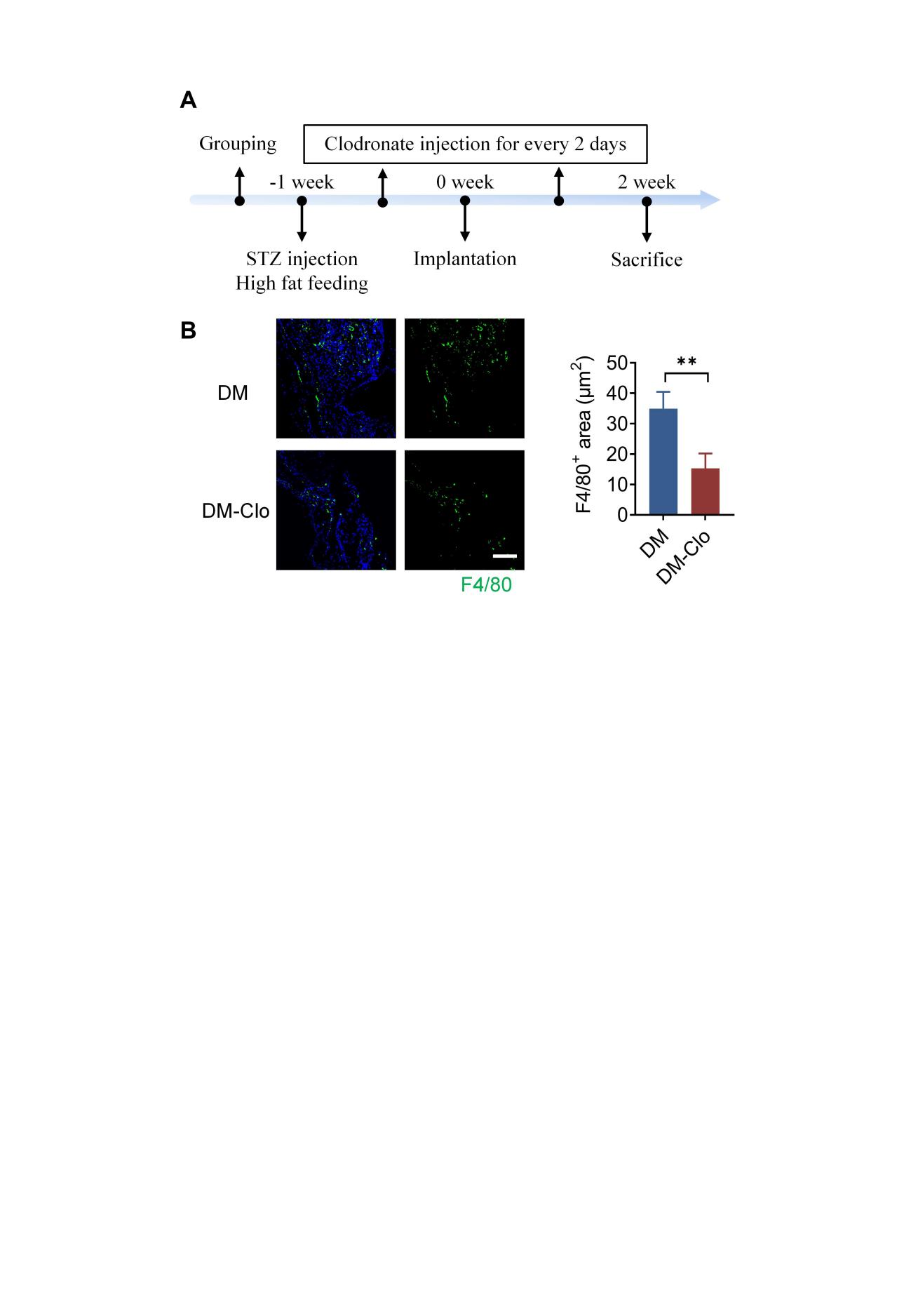


**Fig.SI-5 Preparation and characteristics of macrophage-depleted DM mice.** A) Schematic diagrams of macrophage-depleted DM Mice. B) Representative staining images and quantification of F4/80^+^ macrophages in the skulls of DM mice with or without clodronate treatment (n = 4). ***P* < 0.01.

**SI-6.** Silicon collaborates with macrophages to enhance angiogenesis and nerve regeneration via microvesicles.


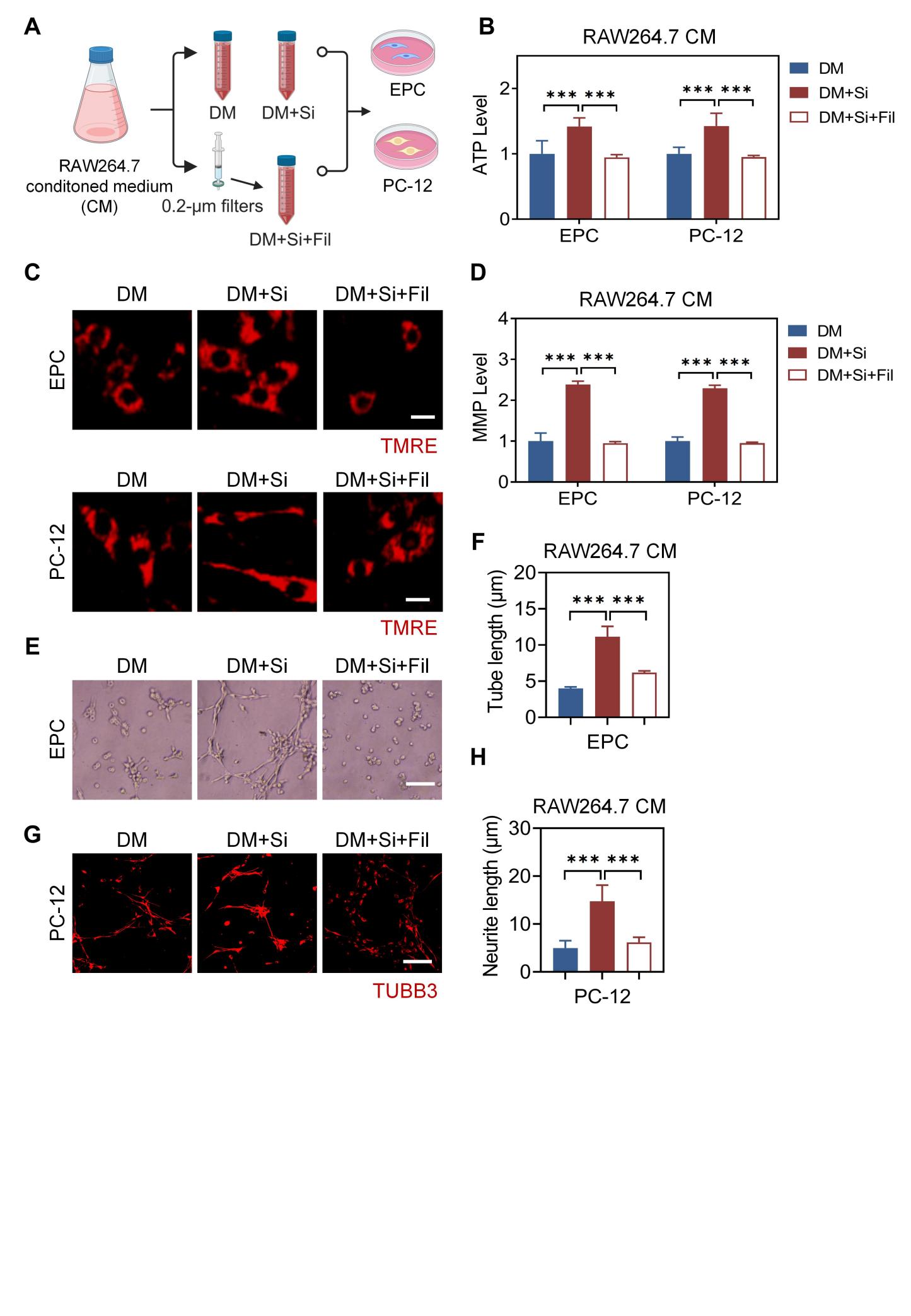


**Fig.6 Silicon collaborates with macrophages to enhance angiogenesis and nerve regeneration via microvesicles.** A) Schematic showing the experimental set-up. B) Quantification of intracellular ATP protection in endothelial progenitor cells (EPC) or PC-12 under indicated treatments (n = 6). C, D) Representative pictures and quantification of mitochondrial membrane potential in EPC or PC-12 under indicated treatments using TMRE intensity (n = 6). Scale bar: 10 μm. E, F) Representative images and quantification of tubular structures formed by EPC under the indicated treatments (n = 6). Scale bar: 100 μm. G, H) Representative images and quantification of neurite length in PC-12 cells under the indicated treatments (n = 6). Scale bar: 100 μm. CM: conditioned medium, DM: diabetic condition, DM+Si: diabetic condition with silicon supplement,. ****P* < 0.001.

**SI-7.** Isolate and identify extracellular vesicles produced by macrophages under DM or DM+Si conditions.


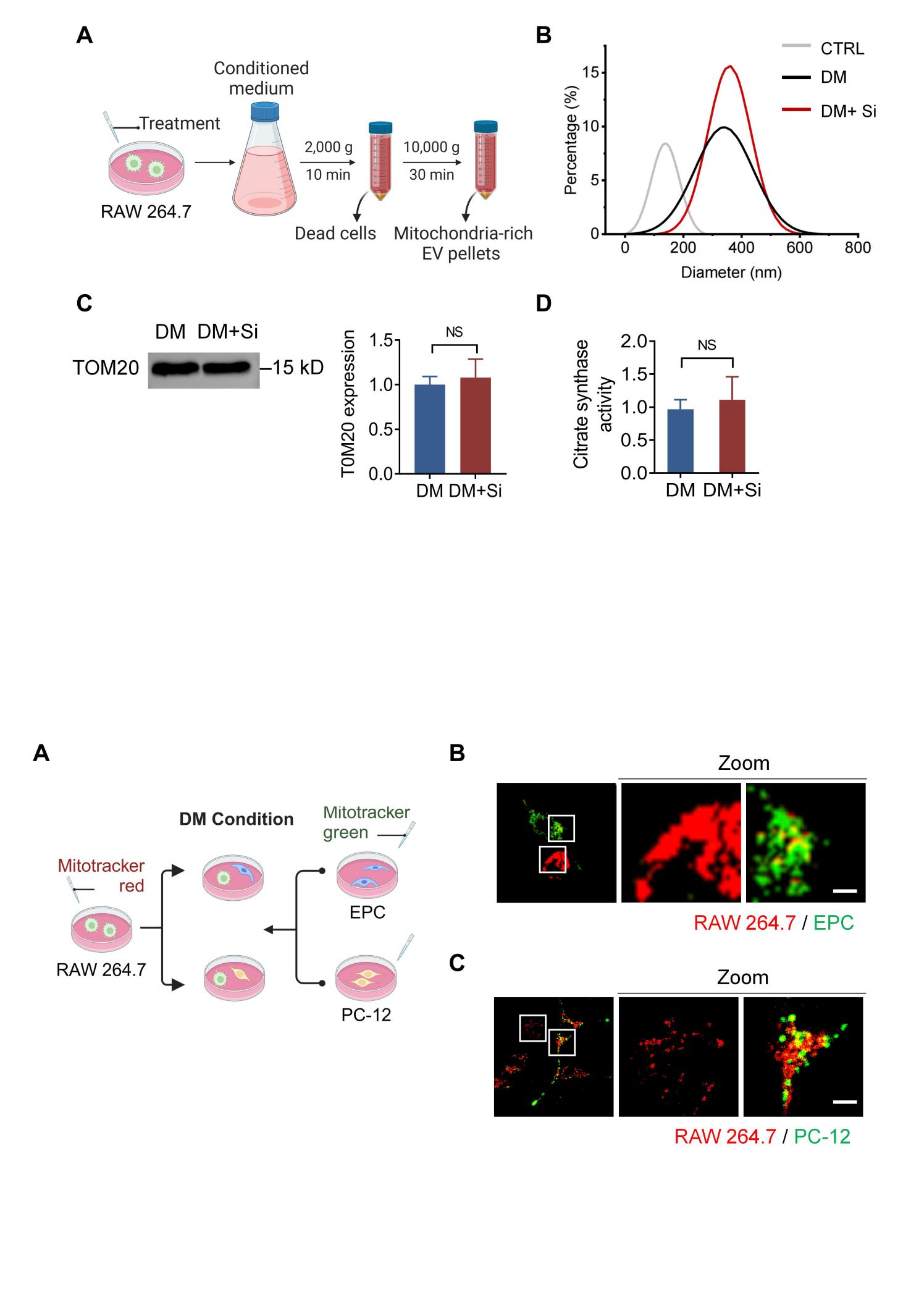


**Fig.SI-7 Isolate and identify extracellular vesicles produced by macrophages under DM or DM+Si conditions.** A) Diagram of extracellular vesicles isolated by gradient centrifugation. B) Particle size analysis of extracellular vesicles. C) Western blot bands and quantitative analysis of mitochondrial marker proteins TOM20 (n = 4). D) Citrate synthase activity in extracellular mitochondria derived from macrophage under DM or DM+Si condition (n = 4).

**SI-8.** Macrophages expel mitochondria through extracellular vesicles under DM condition.

**
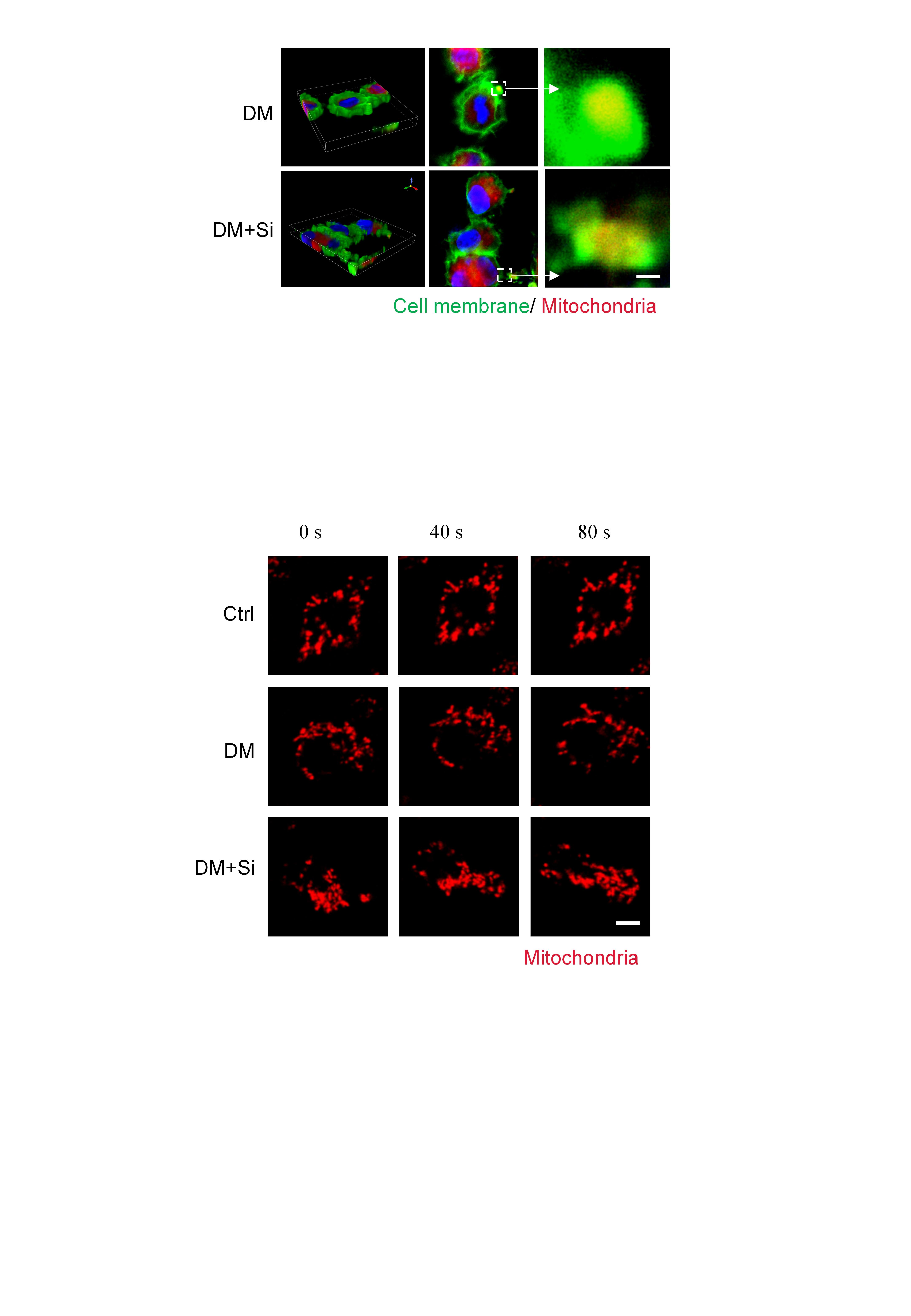
**

**Fig.SI-8 Macrophages expel mitochondria through extracellular vesicles under DM condition.** Representative images of the position relationship between mitochondria and cell membrane of macrophages under different culture conditions. Scale bar: 500 nm.

**SI-9.** Mitochondria released from macrophages transfer to endothelial and neuronal cells.


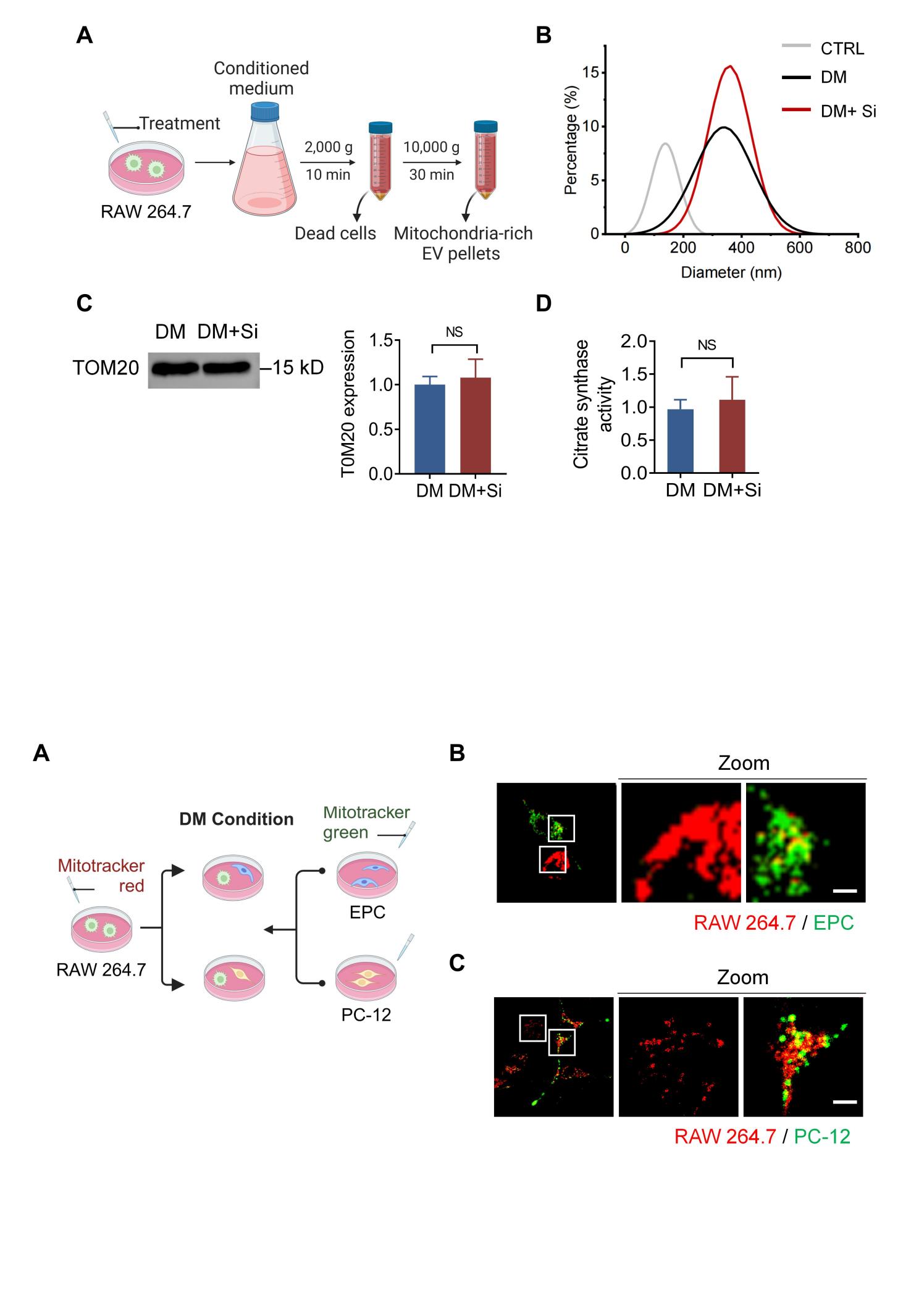


**Fig.SI-9 Mitochondria released from macrophages transfer to endothelial and neuronal cells**. A) Schematic showing the experimental set-up. Mitochondria in RAW 264.7 were stained with MitoTracker Red while those in EPC or PC-12 were stained with MitoTracker Green before co-cultivation. B) Representative pictures of mitochondrial signal in RAW 264.7 and EPC cultured in DM medium. C) Representative pictures of mitochondrial signal in RAW 264.7 and PC-12 cultured in DM medium. Scale bar: 5 μm.

**SI-10.** DM condition changes the mitochondrial movement in macrophages.

**
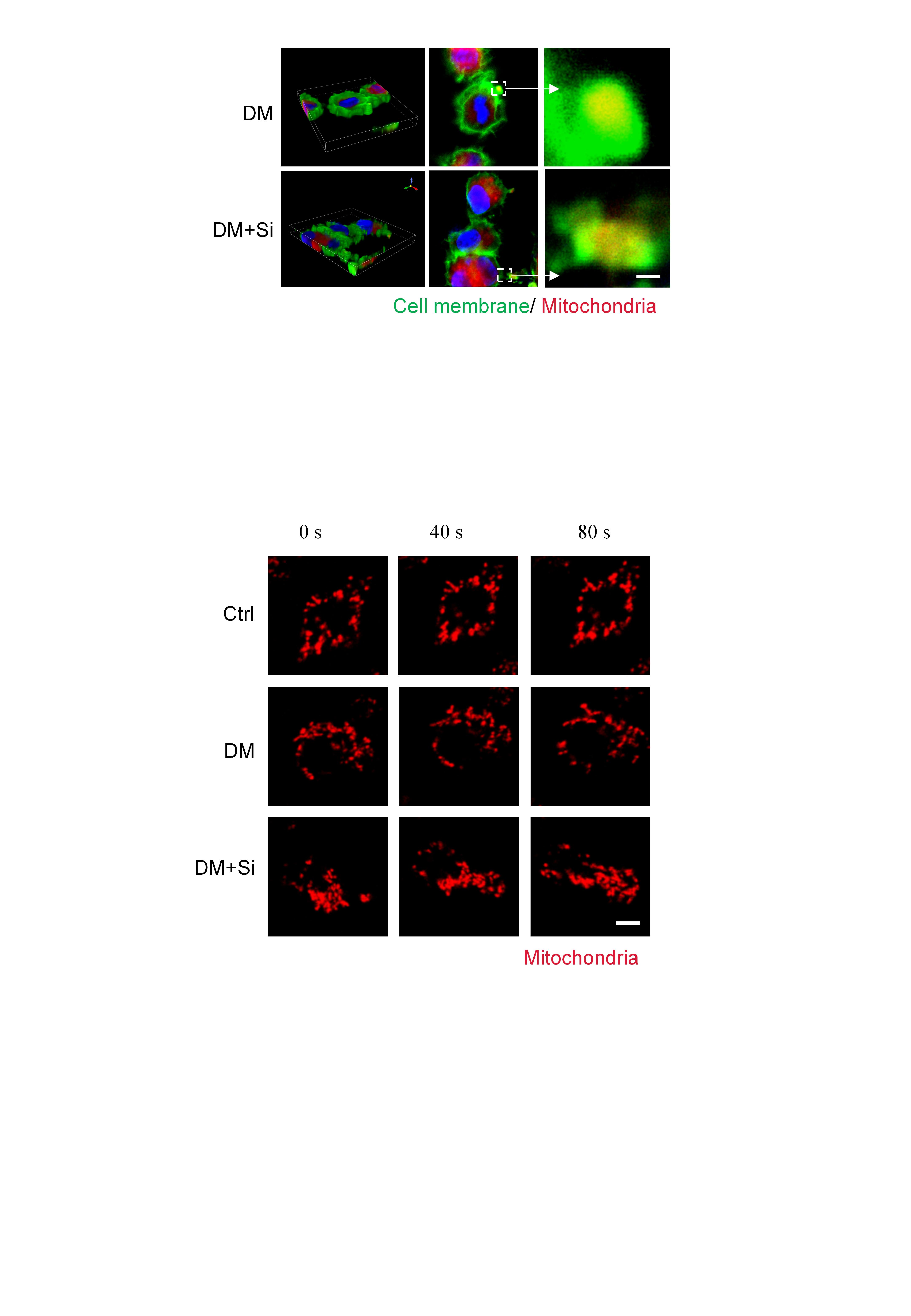
**

**SI-10. DM condition changes the mitochondrial movement in macrophages.** Representative images of mitochondrial movement of macrophages under different culture conditions. Scale bar: 10 μm.

**SI-11.** Effect of macrophage mitochondrial division on mitochondrial transfer under DM condition.


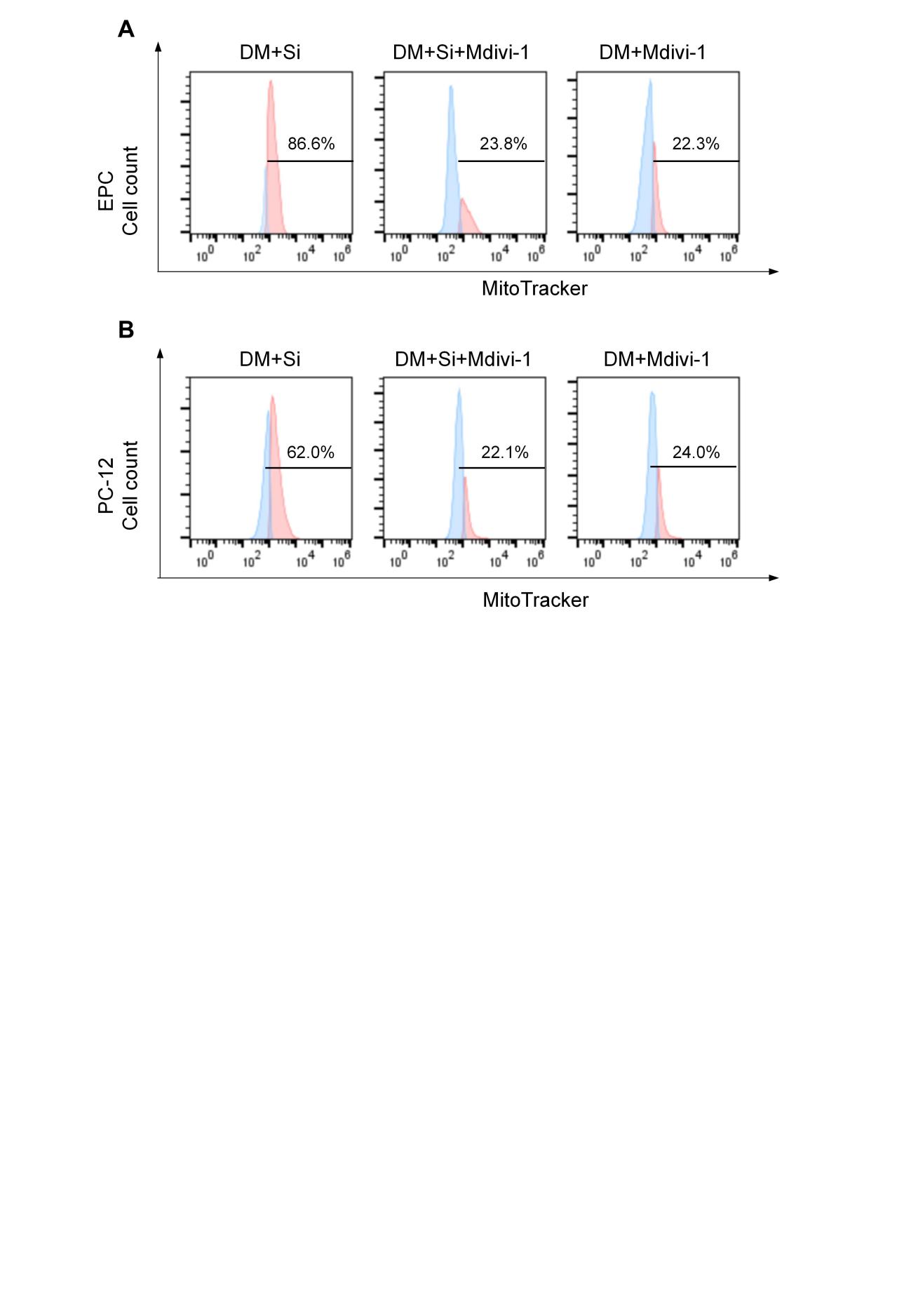


**Fig.SI-11 Effect of macrophage mitochondrial division on mitochondrial transfer under DM condition.** A) Flow cytometry analysis of mitochondrial transfer from macrophages to EPC cells under different culture conditions. B) Flow cytometry analysis of mitochondrial transfer from macrophages to PC-12 cells under different culture conditions.

**Fig.SI-12** Si enhanced the interaction between Mff and Drp1 under induced simulated diabetic condition.


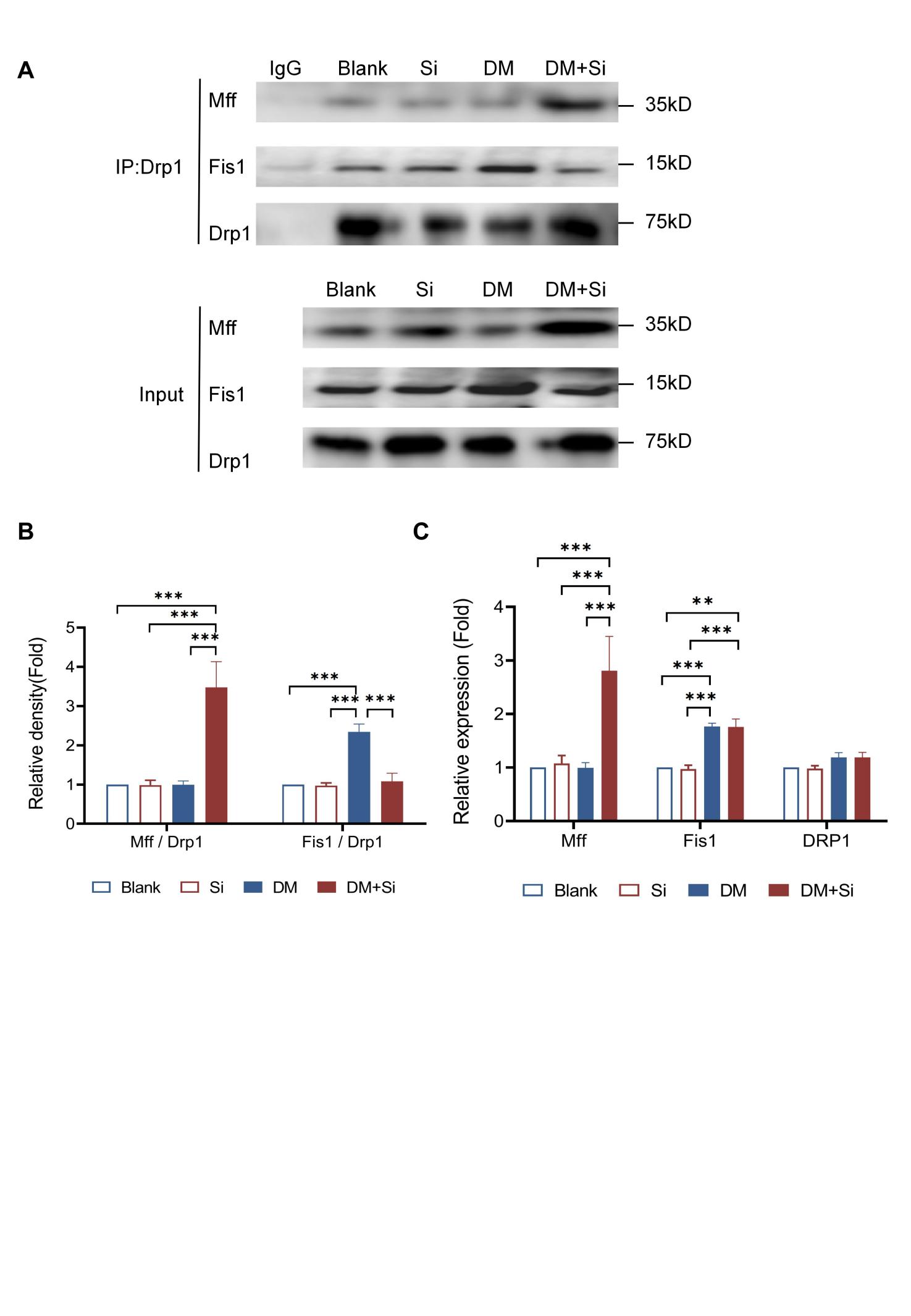


**Fig.SI-12 Silicon enhanced the interaction between Mff and Drp1 under induced simulated diabetic condition.** A) Immunoprecipitation analysis of the Fis1-Drp1 interaction and Mff-Drp1 interaction in RAW264.7 cells. Total cell lysates were subjected to immunoprecipitation (IP) with anti-Drp1 antibodies. The immunoprecipitates were subsequently analyzed by immunoblotting with anti-Mff and anti-Fis1 antibodies. B) The relative amount of Mff and Fis1 that co-immunoprecipitated with Drp1 was determined by normalizing levels (n = 3). C) Expression levels of Drp1, Mff, and Fis1 in the input were determined using western blot (n = 3). Statistical analysis was performed using one-way ANOVA, with significance defined as *P < 0.05, ***P < 0.001.

**SI-13.** P110 reduces mitochondrial reactive oxygen species and improves oxidative stress in macrophages.

**
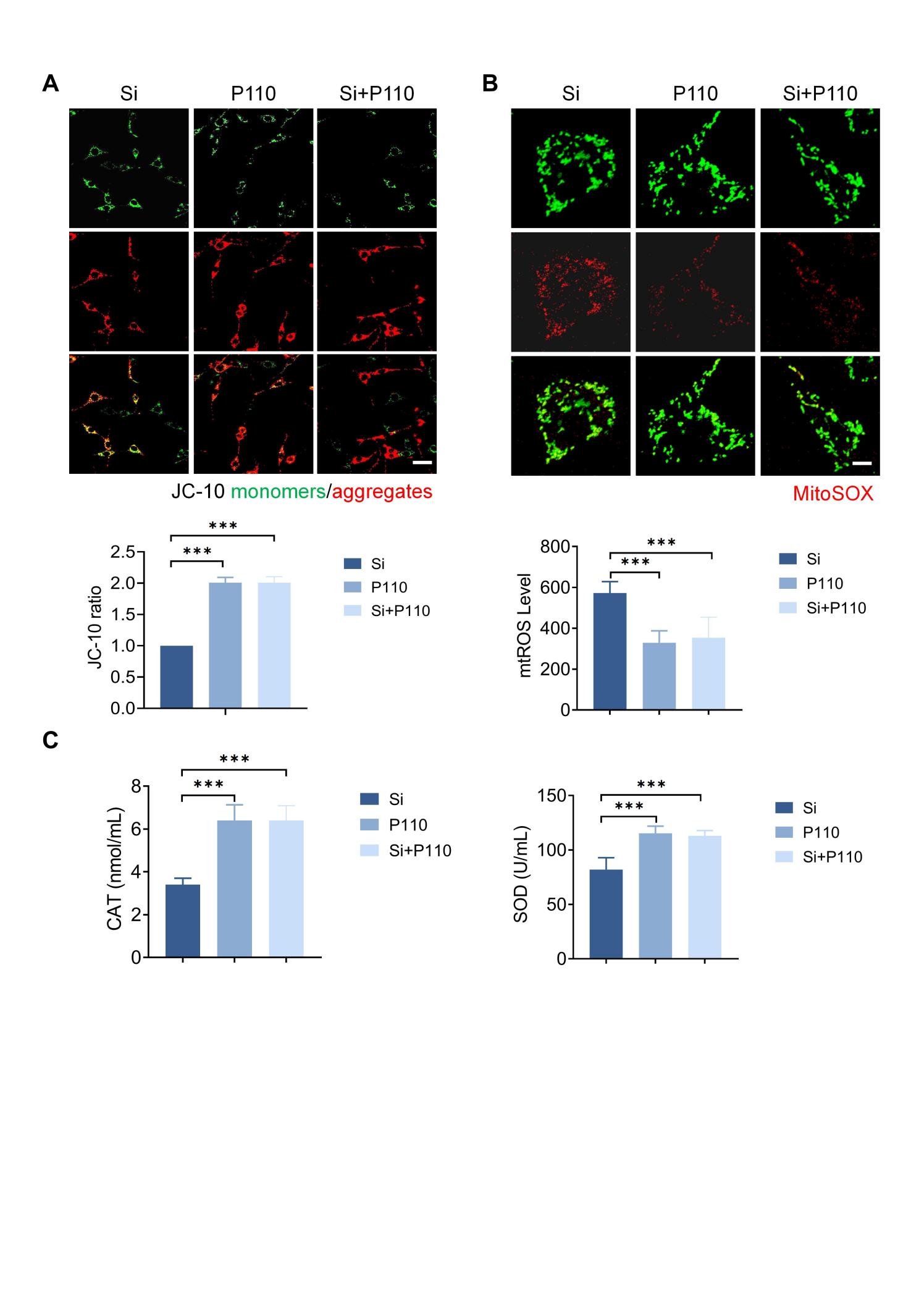
**

**Fig.SI-13** **P110 reduces mitochondrial reactive oxygen species and improves oxidative stress in macrophages.** A) Representative staining images and quantification of JC-10 staining to determine mitochondrial membrane potential in RAW264.7 cells (n = 6). Scale bar: 100 μm. B) Representative staining images and quantification of mtochondrion-specific superoxide (MitoSOX) staining in RAW264.7 cells (n = 6). C) Detection of superoxide dismutase (SOD) and catalase (CAT) in RAW264.7 cells under different conditions (n = 6). Scale bar: 10 μm. ****P* < 0.001.

**SI-14.** Primer sequences used for RT-PCR in the present study.

| **Gene name** | **Forward/ Reverse** | **Sequence （5’-3’）** |
| --- | --- | --- |
| PGC-1α | Forward | GACAATCCCGAAGACACTACAG |
|  | Reverse | AGAGAGGAGAGAGAGAGAGAGA’ |
| NRF1 | Forward | ACCCTCAGTCTCACGACTAT |
|  | Reverse | GAACACTCCTCAGACCCTTAAC |
| TFAM | Forward | GAAGGGAATGGGAAAGGTAGAG |
|  | Reverse | ACAGGACATGGAAAGCAGATTA |
| Mfn1 | Forward | GTTGGAGCGGAGACTTAGCA |
|  | Reverse | TCCGAGATAGCACCTCACCA |
| Opa1 | Forward | GGAAGTCCATGCGCCATT |
|  | Reverse | CACTAAAGACTGGCAGACCTC |
| DRP1 | Forward | CTCTGGCCAATAGAAATGGAACA |
|  | Reverse | TCATCCACGGGTTCACCGTA |
| Fis1 | Forward | GTAAAGGCATCGTGCTGCTC |
|  | Reverse | ACGGCCAGGTAGAAGACGTA |
| Mff | Forward | GCGAATGAGGGTCCCAGAAA |
|  | Reverse | TGTTTTCAGTGCCAGGGGTT |
| GAPDH | Forward | GGGAAGCCCATCACCATCTT |
|  | Reverse | TCGTGGTTCACACCCATCAC |

**Table SI-1.**  Sequences of primers for PCR
